# Coordinated Interferon-γ/Tumor Necrosis Factor Axis Drives Selective Loss of Activated Enteric Glia in Inflammatory Bowel Diseases

**DOI:** 10.1016/j.jcmgh.2026.101827

**Published:** 2026-06-12

**Authors:** Marvin Bubeck, Klara A. Penkert, Heidi Limberger, Miguel González Acera, Christina Plattner, Svenja Ziegler, Anoohya Muppirala, Patrycja Forster, Manuel Jakob, Reyes Gamez-Belmonte, Lena Erkert, Subhash Kulkarni, Claudia Günther, Raja Atreya, Anja A. Kühl, Ahmed N. Hegazy, Kai Hildner, Zlatko Trajanoski, Britta Siegmund, Markus F. Neurath, IBDome Consortium, Imke Atreya, Raja Atreya, Petra Bacher, Christoph Becker, Christian Bojarski, Nathalie Britzen-Laurent, Caroline Voskens, Hyun-Dong Chang, Andreas Diefenbach, Claudia Günther, Ahmed N. Hegazy, Kai Hildner, Christoph S.N. Klose, Kristina Koop, Susanne M. Krug, Anja A. Kühl, Moritz Leppkes, Rocío López-Posadas, Leif S. Ludwig, Clemens Neufert, Markus Neurath, Jay V. Patankar, Christina Plattner, Magdalena S. Prüß, Andreas Radbruch, Chiara Romagnani, Francesca Ronchi, Ashley D. Sanders, Alexander Scheffold, Jörg-Dieter Schulzke, Michael Schumann, Sebastian Schürmann, Britta Siegmund, Michael Stürzl, Zlatko Trajanoski, Antigoni Triantafyllopoulou, Maximilian Waldner, Carl Weidinger, Stefan Wirtz, Sebastian Zundler, Meenakshi Rao, Fränze Progatzky, Dieter Chichung Lie, Christoph Becker, Chiara Romagnani, Leif S. Ludwig, Christoph S.N. Klose, Jay V. Patankar

**Affiliations:** 1 Department of Medicine 1, Universitätsklinikum Erlangen and Friedrich-Alexander-Universität Erlangen- Nürnberg (FAU), Erlangen, Germany; 2 Deutsche Zentrum Immuntherapie (DZI), Friedrich-Alexander-Universitat Erlangen-Nurnberg, Erlangen, Germany; 3 Berlin Institute of Health at Charité - Universitätsmedizin Berlin, Berlin, Germany; 4 Max-Delbrück-Center for Molecular Medicine in the Helmholtz Association (MDC), Institute for Medical Systems Biology (BIMSB), Berlin, Germany; 5 Charité - Universitätsmedizin Berlin, Berlin, Germany; 6 Biocenter, Institute of Bioinformatics, Medical University of Innsbruck, Innsbruck, Austria; 7 Department of Hematology, Oncology, and Tumor Immunology, Charité, Berlin, Germany; 8 Division of Gastroenterology, Department of Pediatrics, Boston Children’s Hospital and Harvard Medical School, Boston, Massachusetts; 9 Department of Microbiology, Infectious Diseases and Immunology, Charité – Universitätsmedizin Berlin, Corporate Member of Freie Universität Berlin and Humboldt-Universität zu Berlin, Berlin, Germany; 10 Division of Gastroenterology, Department of Medicine, Beth Israel Deaconess Medical Center, Boston, Massachusetts; 11 Division of Medical Sciences, Harvard Medical School, Boston, Massachusetts; 12 iPATH.Berlin, Charité - Universitätsmedizin Berlin, Freie Universität Berlin, Humboldt Universität zu Berlin, Berlin, Germany; 13 Department of Gastroenterology, Infectious Diseases and Rheumatology, Charité – Universitätsmedizin Berlin, Corporate Member of Freie Universität Berlin and Humboldt-Universität zu Berlin, Berlin, Germany; 14 The Kennedy Institute of Rheumatology, University of Oxford, Oxford, United Kingdom; 15 Institut für Anatomie, Lehrstuhl für Mikroskopische Anatomie und Molekulare Bildgebung, Friedrich-Alexander-Universität Erlangen- Nürnberg (FAU), Erlangen, Germany; 16 Institute of Medical Immunology, Charité Universitätsmedizin Berlin, Freie Universität Berlin and Humboldt Universität zu Berlin, Berlin, Germany; 17 Deutsches Rheuma-Forschungszentrum Berlin (DRFZ), ein Leibniz Institut, Berlin, Germany

**Keywords:** Enteric Glial Cells, IFN-γ, Inflammatory Bowel Disease, Necroptosis, TNF

## Abstract

**Background & Aims:**

Enteric glial cells regulate gastrointestinal homeostasis and inflammation. Although activated enteric glial cells have been shown to support epithelial and immune balance in preclinical models, their functional status and turnover in inflammatory bowel diseases remain poorly defined. This study aimed to identify enteric glial cell activation markers and assess their susceptibility to cytokine-driven death in inflammatory bowel diseases.

**Methods:**

We analyzed 390 intestinal samples from patients with inflammatory bowel disease using bulk and single-nucleus RNA sequencing and validated findings across public datasets comprising over 1160 patients and 19,000 enteric glial cell transcriptomes. We used multiple mouse models of gut inflammation, reporter-based glial sorting, transcriptomics, and glia-specific *Casp8* deletion to dissect mechanisms of enteric glial cell activation and death. Ex vivo stimulation of sorted enteric glial cells was used to assess cytokine-specific effects.

**Results:**

We identified novel inflammatory bowel disease subtype– and location-specific enteric glial cell activation markers, including osteopontin (*SPP1*), enriched in ulcerative colitis. Single-nucleus and single-cell data revealed that activated enteric glial cell clusters selectively upregulate cell death signatures with inflammatory bowel disease enteric glial cells displaying necroptosis via phosphorylation of mixed lineage kinase domain-like pseudokinase. In mice, acute T helper 1 cell/T helper 17–driven inflammation rapidly induced enteric glial cell activation and necroptosis, impairing intestinal motility. Ex vivo, interferon-γ and tumor necrosis factor costimulation, but not individual cytokines, induced mixed lineage kinase domain-like pseudokinase–dependent necroptosis in enteric glial cells. *Casp8*-deficient enteric glial cells ere hypersensitive to tumor necrosis factor–induced death, confirming a Caspase-8-dependent survival checkpoint.

**Conclusions:**

A proportion of activated enteric glial cells are selectively eliminated in inflammatory bowel disease via cytokine-mediated necroptosis, driven by a coordinated interferon-γ/tumor necrosis factor axis. This process compromises enteric glial support functions and may contribute to inflammatory bowel disease–associated dysmotility. Targeting glial survival may represent a novel therapeutic avenue.

**Summary**

This study identifies a coordinated interferon-γ/tumor necrosis factor axis that triggers selective necroptosis of activated enteric glial cells in inflammatory bowel diseases. This homeostatic failure compromises vital glial support functions and drives gut dysmotility, highlighting glial survival as a promising new therapeutic target.

**What You Need to Know**

**Background**

Enteric glial cells regulate gut homeostasisbut their turnover in inflammatory bowel disease is poorly understood. This study identifies new activation markers and examines glial susceptibility to cytokine-driven death.

**Impact**

Activated inflammatory bowel disease glia express immunomodulatory osteopontin. A coordinated interferon-γ/tumor necrosis factor axis drives necroptosis of activated enteric glia in inflammatory bowel disease. This process impairs glial support functions contributing to disease-associated dysmotility.

**Future Directions**

Targeting glial survival pathways presents a novel therapeutic avenue for inflammatory bowel disease–related dysmotility. Novel markers like osteopontin may serve as surrogate indicators for monitoring glial network integrity and stratifying patient risk.

Inflammatory bowel diseases (IBDs) pose a chronic inflammatory challenge, demanding concerted actions of multiple cells in restoring homeostasis. Enteric glial cells (EGCs) display distinct morphotypes and activation states controlling gut motility, visceral hypersensitivity, and lymphocyte activation.^1^, ^2^, ^3^, ^4^, ^5^ Data from preclinical models has revealed that activated EGC subtypes control epithelial, immune, and neuronal homeostasis during intestinal inflammation. For example, during intestinal inflammation activated EGC express: (1) major histocompatibility complex class 2 (MHC-II), inducing tolerogenic T-cells^6^; (2) Wnt ligands regulating epithelial regeneration^3^; (3) CSF1 promoting monocyte recruitment^2^; and (4) CXCL10 promoting helminth clearance.^7^

Despite this, the translation of these findings in the context of IBDs has been limited largely due to a lack of consensus on what constitutes activated EGCs. Through multiple studies in mouse models, glial fibrillary acidic protein (GFAP) has emerged as a bona fide activation marker for EGCs. However, its expression and specificity in human EGCs is highly contested. Furthermore, the status of S100 calcium-binding protein B (S100B), another prevailing activation marker of human EGCs, was also challenged recently.^8^ The S100B regulates survival and can act as a damage-associated molecular pattern, and hence its elevation has been linked with EGC activation. Another broadly expressed maker for EGCs is proteolipid protein 1 (PLP1), with an established role in myelination in the peripheral nervous system. However, its regulation during gut inflammation and its functions in the nonmyelinated enteric nervous system (ENS) are poorly defined, with recent studies pointing to a role in the regulation of gut motility in mice.^4^ This is reflected in the disparity in previous reports, some indicating EGC elevation, whereas other showing EGC apoptosis and reduction in EGC frequency in IBDs.^9^, ^10^, ^11^, ^12^, ^13^, ^14^ This is fueled by challenges in analyzing all EGC subsets from patient samples, because translational research is dominated by data derived from mucosal biopsies, which miss out on myenteric and longitudinal muscle EGCs, limiting a clearer insight on EGC activation markers in IBDs.

An early consequence of gut inflammation is dysmotility, induced in acute conditions and sustained by dysbiotic microbial insults. This can trigger enteric neuronal pyroptosis and, as we have recently shown, neuronal, but not EGC ferroptosis.^15^, ^16^, ^17^ It is currently unknown whether and how EGCs may evade programs of cytokine-mediated pathologic cell death such as necroptosis and how this affects EGC turnover in IBDs. This is especially important in the context of anti–tumor necrosis factor (TNF) therapy, TNF being an upstream inducer of necroptosis, where few studies in experimental colitis have investigated neuroglia protection after anti-TNF treatment.^18^

Based on these considerations, we took a highly integrated approach to first identify EGC activation markers in IBD types, comparing biopsy and resection tissues in bulk, single-nucleus (sn), and single-cell (sc) transcriptomes from patients with IBDs. We uncovered that, specifically, the activated EGC subsets display elevated expression of cell death pathway signatures. Analysis of sorted EGCs revealed a combined requirement of TNF and interferon- γ (IFN-γ) in mediating EGC necroptosis. Our study provides novel EGC-enriched activation markers elevated in IBDs, which regulate EGC-immune and EGC-epithelial homeostasis and highlight TNF- and IFN-γ–mediated activated EGC necroptosis as a component feature in helper T cell 1 (Th1)/helper T cell 17 (Th17)-driven intestinal inflammation. Further studies aimed at investigating EGC turnover, cellular states, and EGC-immune and -epithelial crosstalk are necessary for teasing out the translational impact of crucial EGC functions in IBDs.

## Results

### Identification of Enteric Glial Cell Activation Markers Across Inflammatory Bowel Diseases

**Figure 1:**
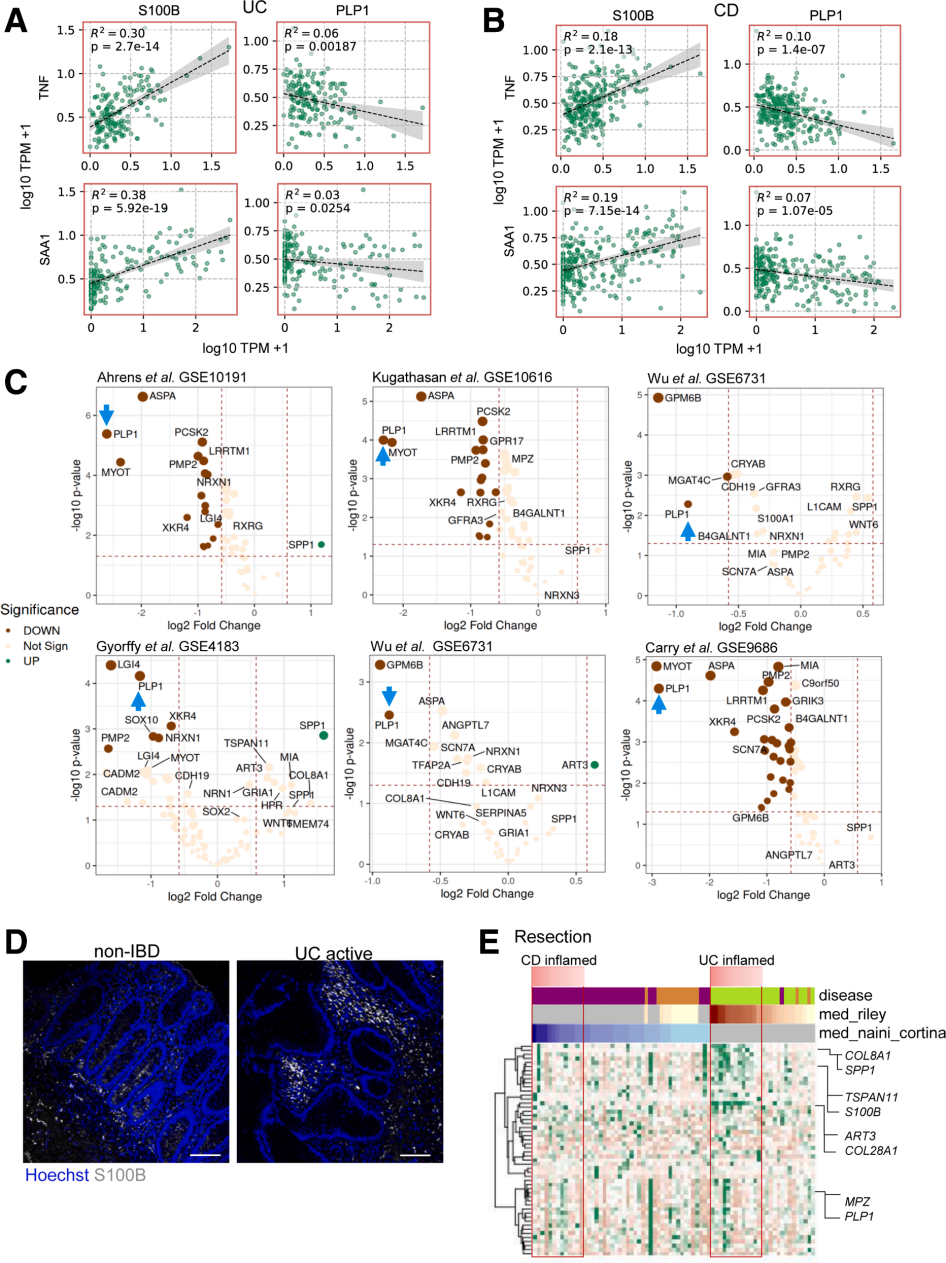
**Correlation of canonical and novel EGC markers on in-house and replication cohort.** (*A* and *B*) Correlation plots for the patient-wise transcript expression levels (each *dot* represents levels from 1 patient) of the indicated inflammation markers (*TNF*, *SAA1*) vs canonical EGC markers *S100B*, and *PLP1* across all samples in the IBDome cohort irrespective of sampling procedure or degree of inflammation for UC (*A*) and CD (*B*). The square of the Pearson’s *r* and the associated *P* value are denoted on the plots. (*C*) Expression levels of EGC-enriched genes obtained as per scheme in Figure 2*A*, on 6 indicated publicly available microarray datasets from patients with IBD showing consistent reduction of *PLP1* (*arrows*). (*D*) Representative photomicrographs of immunostained tissues showing high levels of mucosal S100B-positive cells from patients with active UC compared with non-IBD CTRLs. (*E*) Patient-wise z-score heatmaps of the same EGC genes identified in Figure 3*A*, grouped by median histologic severity scores for resections. Rows clustered by expression using Ward’s method. The *red rectangles* outline the inflamed patients.

**Figure 2:**
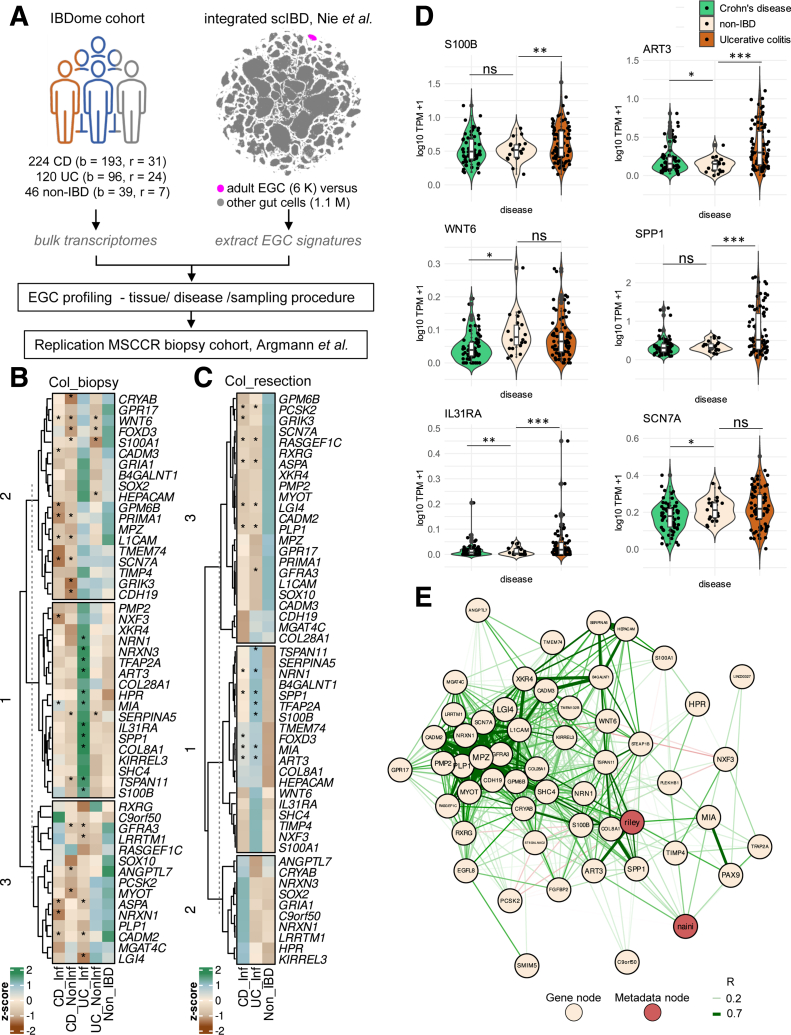
**IBD subtype, sampling procedure, and inflammation-associated enteric glia signatures.** (*A*) Overview of the IBDome cohort, showing patient numbers by sampling type (b = biopsy, r = resection), EGC-enriched gene signature extraction from integrated sc IBD transcriptomes,^19^ and replication strategy using the MSCCR cohort.^20^ (*B* and *C*) Heatmaps of mean z-scores for EGC genes identified from (*A*) on colonic biopsies (*B*) and resections (*C*). *Asterisks* denote *P* < .05 (Mann–Whitney test vs non-IBD). (*D*) Violin plots for normalized counts of the indicated EGC-enriched genes in the IBDome cohort across the indicated IBD types or non-IBD CTRLs. *Asterisks* indicate the Mann–Whitney *U* test *P* values, ∗*P* < .05, ∗∗*P* < .01, ∗∗∗*P* < .001, ns, not significant, between indicated groups. (*E*) Network plot where nodes are genes (*beige*) or histology scores (*red*) (riley = modified median Riley score for UC and naini = modified median Naini Cortana score for CD) from colonic biopsy samples of the IBDome cohort. Edge thickness represents Pearson’s *r* with edge color indicating direction of correlation (*green* = positive, *red* = negative). Correlated nodes cluster together.

An initial analysis of resection and mucosal biopsy data from transcriptomes from patients with IBD (IBDome cohort) and previous microarray datasets from patients with IBDs revealed a divergent correlation of the canonical EGC markers *S100B* and *PLP1* against inflammation markers *TNF* and *SAA1* (Figure 1*A–D*). To identify IBD-associated EGC-specific markers, we leveraged the scIBD atlas of over 1.1 million IBD sc transcriptomes. Comparing these sc expression profiles of EGCs vs all other cells, the top IBD-associated EGC markers were extracted and tracked across the bulk transcriptomes of our IBDome patient cohort and the Mount Sinai Crohn’s and Colitis Registry (MSCCR). This enabled comparison of IBD-associated, EGC-specific markers across disease types, tissues, and sampling procedures (Figure 2*A*).^19^^,^^20^

**Figure 3:**
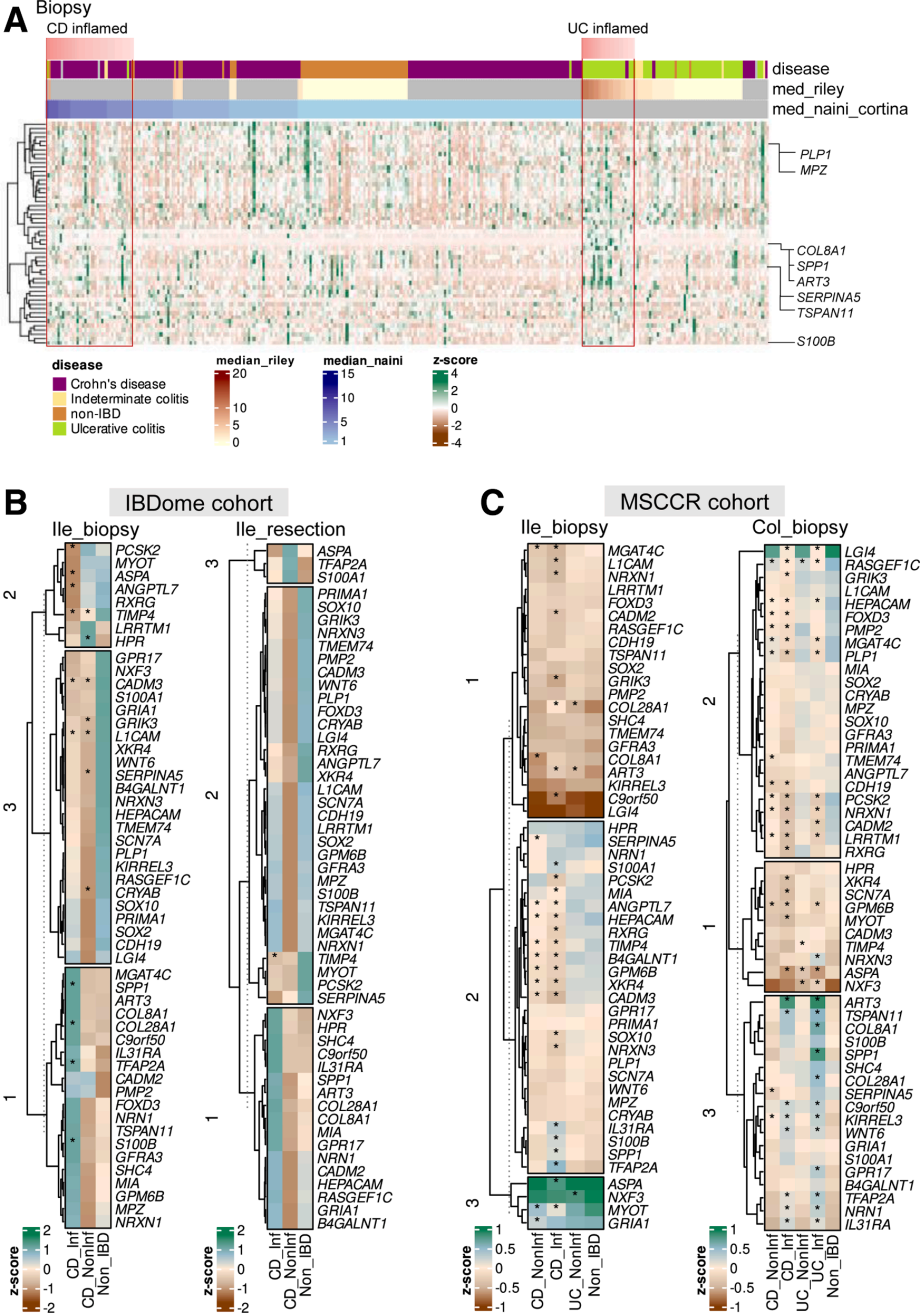
**Correlation of EGC markers on in-house and replication cohort.** (*A*) Patient-wise z-score heatmaps of the same EGC genes identified in Figure 2*A*, grouped by median histologic severity scores for biopsies. Rows clustered by expression using Ward’s method. The *red rectangles* outline the inflamed patients (riley = modified median Riley score for UC and naini = modified median Naini Cortana score for CD). (*B* and *C*) Heatmaps comparing mean z-scores calculated from normalized counts for the EGC-enriched genes identified in Figure 2*A*, across patients with IBD from the IBDome (*B*) and the MSCCR cohorts (*C*) from ileal (Ile_) and colonic (Col_) biopsies or resection samples. *Asterisks* indicate *P* values <.05 from the Mann–Whitney *U* test applied on normalized counts.

Applying the EGC enriched list from Figure 2*A* to the data from the IBDome samples revealed several new inflammation-associated EGC markers in colonic biopsies and resection samples of patients with inflamed ulcerative colitis (UC) (Figure 2*B* and *C*). A number of these EGC markers were associated with inflammation status, irrespective of sampling procedure, although overall z-scores remained higher in the biopsy vs resection samples, implying a differential magnitude of regulation in mucosal and myenteric EGC (Figures 2*B* [biopsy] and *C* [resection]). Unlike that for UC, the overall z-scores obtained for patients with inflamed Crohn’s disease (CD) for these EGC markers did not segregate by histologic inflammation score across the patient level analysis, irrespective of the sampling procedure or tissue (Figures 1*E* and 3*A*). This indicated a strong involvement of EGC adaptations affected in UC but not in CD.

Among the markers impacted was *SPP1*, which was upregulated in inflamed UC, but not CD colon biopsies, and correlated tightly with modified median Riley scores on the IBDome cohort, was concordant in the resections, and was replicated on the MSCCR cohort (Figures 2*B–D* and 3*C*). The expression of *ART3 was* also upregulated in inflamed UC and CD colonic biopsies and resections and correlated with the canonical activation marker *S100B* (Figures 2*B–D*, 3*B* and *C*). Some markers were specifically altered in inflamed CD colonic biopsies (eg, SCN7A, which was downregulated in the inflamed CD colon biopsy samples from the IBDome and the MSCCR cohorts, but not in resection or ileal samples) (Figures 2*B–D*, 3*B* and *C*). Based on their expression across all samples from our IBDome cohort, correlations could be established between the expression of the identified EGC markers vs the respective histologic inflammation scores. This was visualized for colonic biopsy samples in the form of a correlation network identifying new EGC activation markers *SPP1* and *ART3*, which correlated closely with the Riley score for UC and weakly with the Naini Cortana score for CD, again reflecting the strong CD vs UC disparity in EGC activation (Figure 2*E*). Interestingly, this strong UC vs CD disparity in colonic biopsy samples observed in the IBDome cohort was recapitulated for several markers, including *SPP1*, on the colonic biopsies of the MSCCR cohort (Figure 3*C*). Furthermore, the inflammation-associated upregulation of *SPP1* was also conserved in the ileal biopsies of patients with CD between the IBDome and the MSSCR cohorts (Figure 3*B* and *C*). These findings highlight new inflammation-associated EGC markers, including the immunomodulatory *SPP1* in biopsy and resection samples as well as in CD and UC.

### Single-Nuclei RNA Sequencing Identifies Inflammatory Bowel Disease–Associated Enteric Glial Cell Clusters With Elevated Cell Death Signature

**Figure 4:**
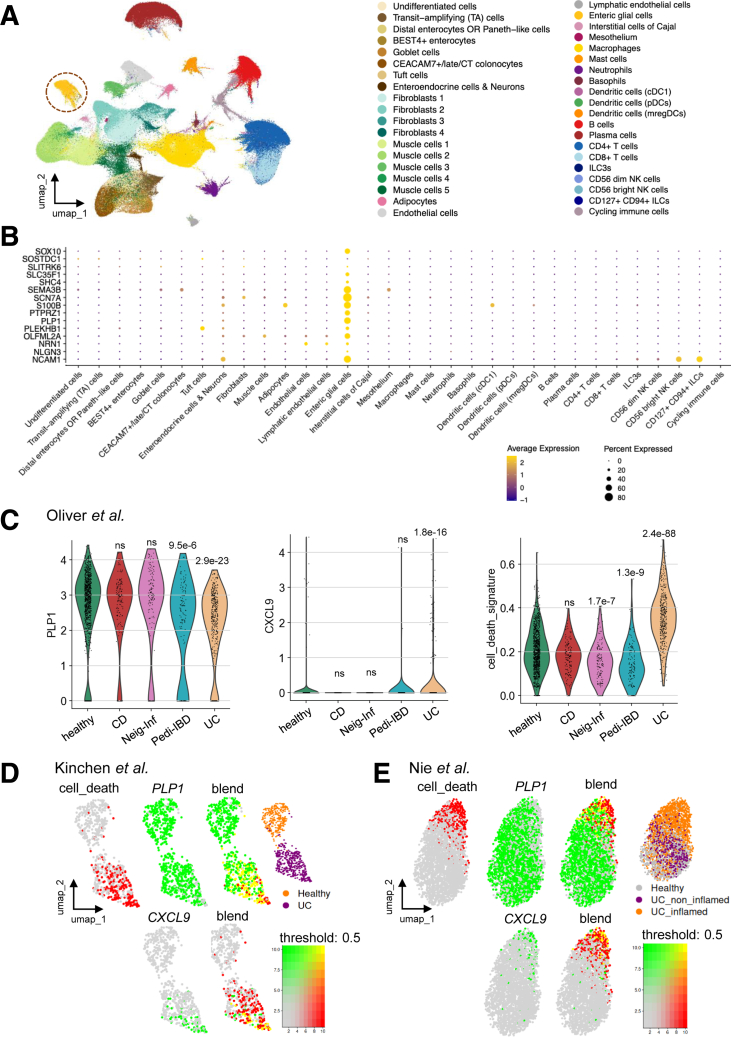
**Transcriptomes from IBD single EGCs and single EGC nuclei reveal cluster and IBD subtype-specific induction in cell death and activation signatures.** (*A*) Uniform manifold approximation and projection (UMAP) plots from snRNA-seq from colonic full thickness formalin-fixed and paraffin-embedded tissue sections from patients with IBD and CTRLs. The EGC cluster is *circled with dashed line*. (*B*) Dot plot showing the identification of the EGC cluster using glia-enriched markers. (*C*) Violin plots showing disease type–wise quantification of indicated genes or pathway signatures reanalyzed from the dataset reported by Oliver et al.^21^ Neig-Inf, neighboring inflamed; Pedi-IBD, pediatric IBD. The numbers on the plots indicate false discovery rate–corrected *P* values derived from the 2-tailed Mann–Whitney *U* test vs healthy controls. ns, not significant. (*D* and *E*) Thresholded expression levels for the indicated genes and for cell death signature together with their expression blend showing UC-specific elevation in interferon target *CXCL9,* and of the cell death pathway signature from EGCs on the Kinchen et al^22^ scRNA-seq dataset (*D*) and from the integrated IBD scRNA-seq dataset by Nie et al^19^ (*E*).

**Figure 5:**
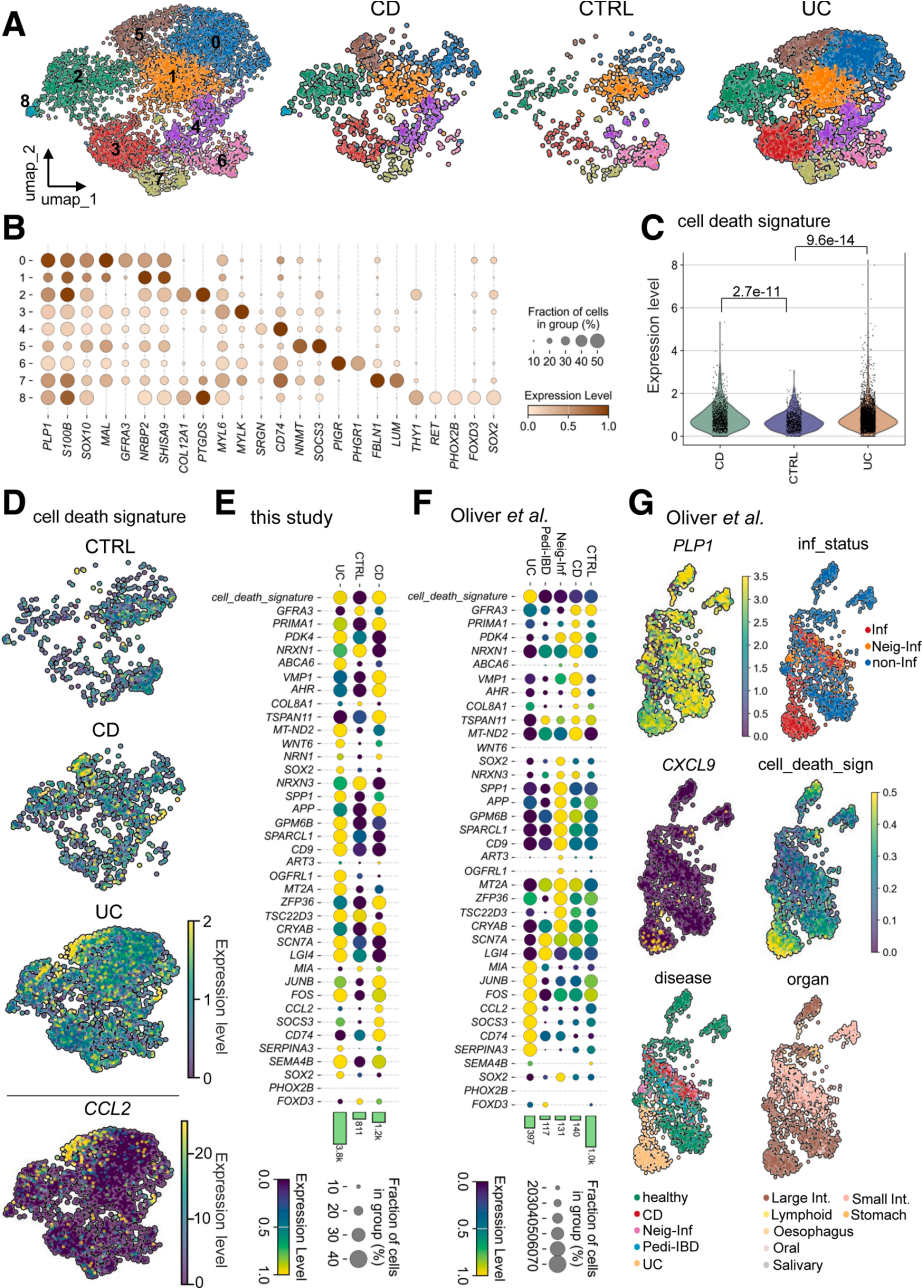
**IBD-associated EGC clusters show distinct activation markers and elevated cell death signature.** (*A*) Uniform Manifold Approximation and Projection (UMAP) of single-nucleus EGC transcriptomes clustered by IBD subtype (UC, CD) and non-IBD CTRLs. (*B*) Dot plot of canonical and cluster-enriched EGC markers. (*C*) Violin plot showing cell death signature expression across IBD subtypes; false discovery rate–adjusted *P* values from Mann–Whitney *U* test. (*D*) UMAP of scaled cell death signature by IBD subtype and bottom that of *CCL2*. (*E* and *F*) Dot plots of selected gene and pathway expression from snRNA-seq data of the IBDome cohort (*E*) and those from the Oliver et al^21^ dataset (*F*). Bar height reflects cell counts. (*G*) UMAPs from Oliver et al^21^ dataset showing EGC gene/pathway expression and cluster annotations. Inf, inflamed; Int, intestine; Neigh-Inf, neighboring inflamed; non-Inf, non-inflamed; Pedi-IBD, pediatric IBD.

To analyze IBD-associated EGC populations more comprehensively, we performed single-nuclear RNA sequencing (snRNA-seq) from full-thickness formalin-fixed, paraffin-embedded tissue sections from the samples in our IBDome patient cohort and non-IBD controls (Figure 4*A*). After quality filtering, we identified 5774 EGC nuclei, defined by expression of canonical EGC markers from previous studies (Figure 4*B*).^7^^,^^23^, ^24^, ^25^, ^26^ Subclustering analysis revealed 9 transcriptionally distinct EGC clusters (Figure 5*A* and *B*). Clusters 0, 2, 3, 4, 5, and 6 showed robust changes between the non-IBD control (CTRL) and the IBD EGCs. Among these, all except cluster 6 were increased in IBDs, whereas cluster 6 was specifically reduced in CD. Clusters 4 and 5 had the most dramatic over-representation in IBD samples and were marked by the expression of *SRGN* and *CD74* (cluster 4) and *NNMT* and *SOCS3* (cluster 5), consistent with previously described reactive or activated glial states.^27^^,^^28^ Notably, the proportion of *PLP1*-expressing EGCs was reduced in these clusters, consistent with the trend observed in bulk transcriptomes and microarray data (Figure 1*A–C*). While analyzing these reactive/ activated glia clusters, we observed that these clusters showed an elevation in several cell death–related markers. To explore this further, we quantified expression of a curated cell death pathway signature (Table 1) across EGC clusters and disease subtypes. This analysis revealed significantly elevated cell death scores in both UC and CD, particularly within clusters 4 and 5, which also showed increased expression of the proinflammatory chemokine *CCL2* (Figure 5*C–E*), hinting that their expansion may be balanced with ongoing cell death.

**Table 1:** Combined Cell Death Pathway Genes for Mouse and Human Used to Map Cell Death Signature on scRNA-seq and snRNA-seq Datasets

Mouse	Human
Aifm1	AIFM1
Anxa1	ANXA1
Apaf1	APAF1
Bad	BAD
Bax	BAX
Bcl2	BCL2
Bgn	BGN
Birc2	BIRC2
Birc3	BIRC3
Casp1	CASP1
Casp3	CASP10
Casp10	CASP3
Casp4	CASP4
Casp6	CASP6
Casp7	CASP7
Casp8	CASP8
Cyld	CYLD
Egr3	EGR3
Emp1	EMP1
Etf1	ETF1
F2r	F2R
Fas	FAS
Fasl	FASLG
Gch1	GCH1
Hgf	HGF
Ier3	IER3
Ifnar1	IFNAR1
Ifnar2	IFNAR2
Ifngr1	IFNAR3
Il1b	IFNGR1
Il1rap	IL1B
Il6	IL1RAP
Irf1	IL6
Isg20	IRF1
Lef1	ISG20
Mlkl	LEF1
Nfkb1	MLKL
Nfkbia	NFKB1
Ntrk1	NFKBIA
Pdgfrb	NTRK1
Pmaip1	PDGFRB
Psen2	PMAIP1
Rela	PSEN2
Ripk1	RELA
Ripk3	RIPK1
Smpd1	RIPK3
Sod2	SMPD1
Tap1	SOD2
Tnfrsf10b	TAP1
Tnfrsf1a	TNFRSF10A
Tnfrsf12a	TNFRSF12A
Tradd	TNFRSF1A
Traf2	TNFRSF10B
Wee1	TRADD
Xiap	TRAF2
Zbp1	WEE1
	XIAP
	ZBP1

scRNA-seq, single-cell RNA sequencing; snRNA-seq, single-nuclear RNA sequencing.

Additionally, several IBD-associated EGC markers identified earlier were recapitulated in the snRNA-seq dataset, most notably *SPP1,* implicated in EGC-immune crosstalk. Moreover, *NRXN1* and *GFRA3*, genes associated with glia-neuron interactions, were significantly downregulated in both our bulk datasets and snRNA-Seq, suggesting that mucosal EGCs may lose neuroregulatory characteristics during gut inflammation. Other broadly immune-related genes (*FOS*, *CCL2*, *SOCS3*) were also upregulated in the activated EGC, although they are shared by other cells and did not appear in our initial marker list, which was focused on analyzing EGC-specific activation markers (Figure 5*E*).

To validate these observations, we reanalyzed 1797 single EGC transcriptomes from the dataset by Oliver et al^21^ (Figure 5*F*). This dataset has a rich annotation for disease subtypes, and several expression patterns overlapped with our findings, including elevated cell death scores and increased expression of *SPP1*, *ART3*, *CCL2*, *SOCS3*, and *FOS* (Figure 5*F*). Importantly, *PLP1* expression was significantly reduced in inflamed UC EGCs (Figures 4*C* and 5*G*), mirroring our bulk transcriptome results. These same clusters also exhibited elevated *CXCL9* expression, an IFN-γ–responsive gene, consistent with findings from Progatzky et al,^7^ and showed increased cell death signature expression.

**Figure 6:**
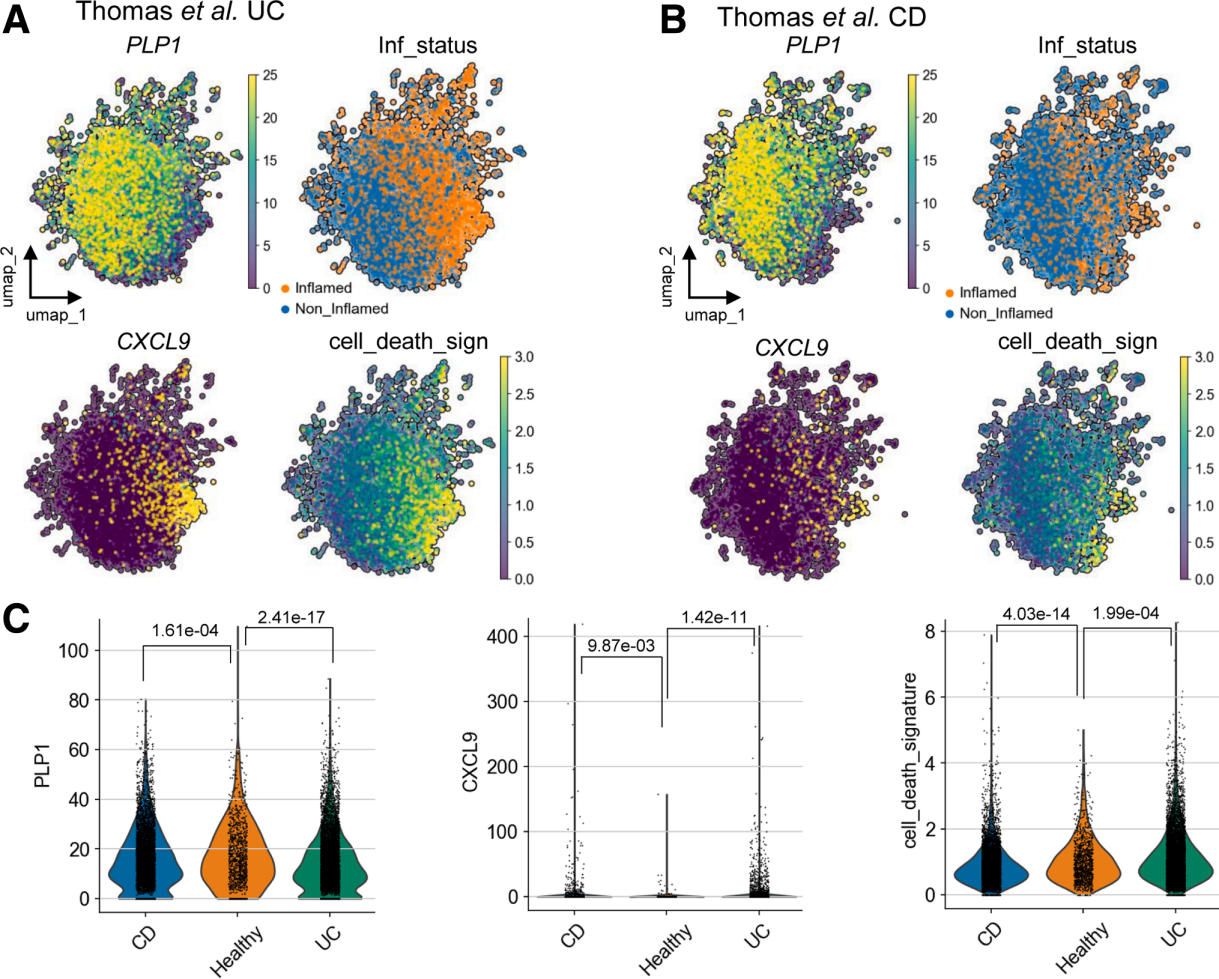
**Transcriptomes from IBD single EGCs reveal induction in cell death and activation signatures.** (*A* and *B*) Uniform Manifold Approximation and Projection (UMAP) plots showing the expression levels of indicated genes and that of the cell death signature along with the inflammation status per cell (Inf_status) from UC (*A*) and CD (*B*) from the single EGC transcriptomes reanalyzed from the dataset by Thomas et al.^29^ (*C*) Violin plots showing quantitative difference for indicated genes and cell death pathway signature reanalyzed from the Thomas et al^29^ dataset shown in (*A*) and (*B*) above. The numbers on the violin plots indicate false discovery rate–corrected *P* values derived from the 2-tailed Mann-Whitney *U* test vs healthy CTRLs.

We further confirmed these trends on 2 independent single-EGC transcriptomic datasets. In the studies by Kinchen et al ^22^ and Nie et al,^19^ specifically, the inflamed EGCs showed similar increases in *CXCL9* and cell death pathway expression, along with *PLP1* downregulation (Figure 4*D* and *E*). Finally, analysis of 13,440 single EGC transcriptomes from Thomas et al ^29^ confirmed upregulation of *CXCL9* and necroptosis signatures, along with reduced *PLP1* in inflamed EGCs from patients with UC and patients with CD (Figure 6*A–C*).

Taken together, these data demonstrate that IBD-associated inflammation, particularly in UC, is linked to the emergence of reactive EGC clusters marked by elevated inflammatory and cell death pathway signatures, loss of neuroregulatory features, and reduced *PLP1* expression, suggestive of cell death induction as a convergent stress phenotype among activated EGC in IBDs.

### Rapid Induction of Enteric Glial Cell Activation and Cell Death in Response to T Helper 1/T Helper 17–Driven Small Intestinal Inflammation

**Figure 7:**
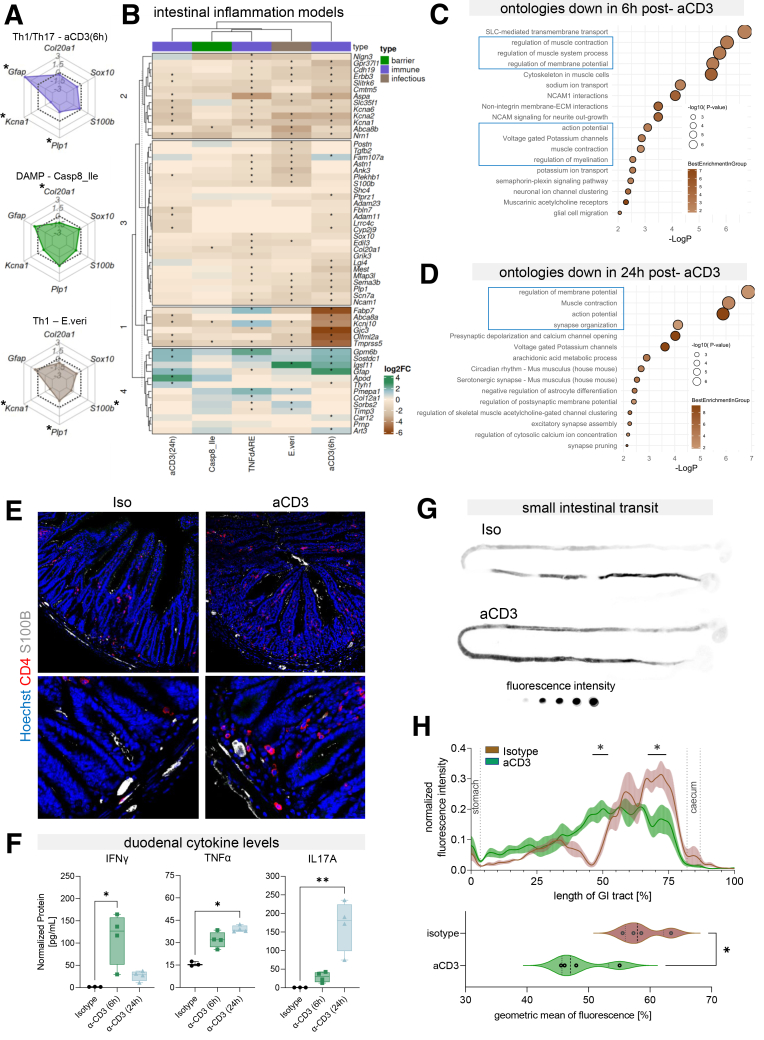
**Th1/Th17-driven intestinal inflammation rapidly alters EGC homeostasis and gut motility.** (*A*) Radar plots showing log2 fold changes of key EGC genes across inflammation models: aCD3 (T cell-driven), Casp8_Ile (epithelial Casp8 deletion), and E veri (infection-induced).^32^ Asterisks denote *P* < .05 (DESeq2, Wald test). (*B*) Heatmap of EGC-enriched genes identified from sorted EGCs^7^ across same models, including TNFdARE^33^; 6 hours/24 hours indicate time post-aCD3. Rows clustered using Ward method. Asterisks denote *P* < .05 (DESeq2, Wald test) (*C* and *D*) Downregulated gene ontologies in the aCD3 model at 6 hours (C) and 24 hours (D); *blue boxes* indicate significant enrichment. (*E*) Representative jejunal sections (6 hours post-aCD3) showing CD4^+^ cells adjacent to S100B^+^ plexuses. (*F*) Cytokine levels in duodenal tissue (Iso vs aCD3); n = 3–4 per group, analysis of variance with Kruskal-Wallis test. (*G* and *H*) Gut motility assessment: (*G*) representative images of dye transit in the gut tissues, and (*H*) normalized intensity (*top*) and geometric mean fluorescence (*bottom*); n = 4 per group, 2-way analysis of variance with Tukey’s test (*top*) and Mann–Whitney (*bottom*).

**Figure 8:**
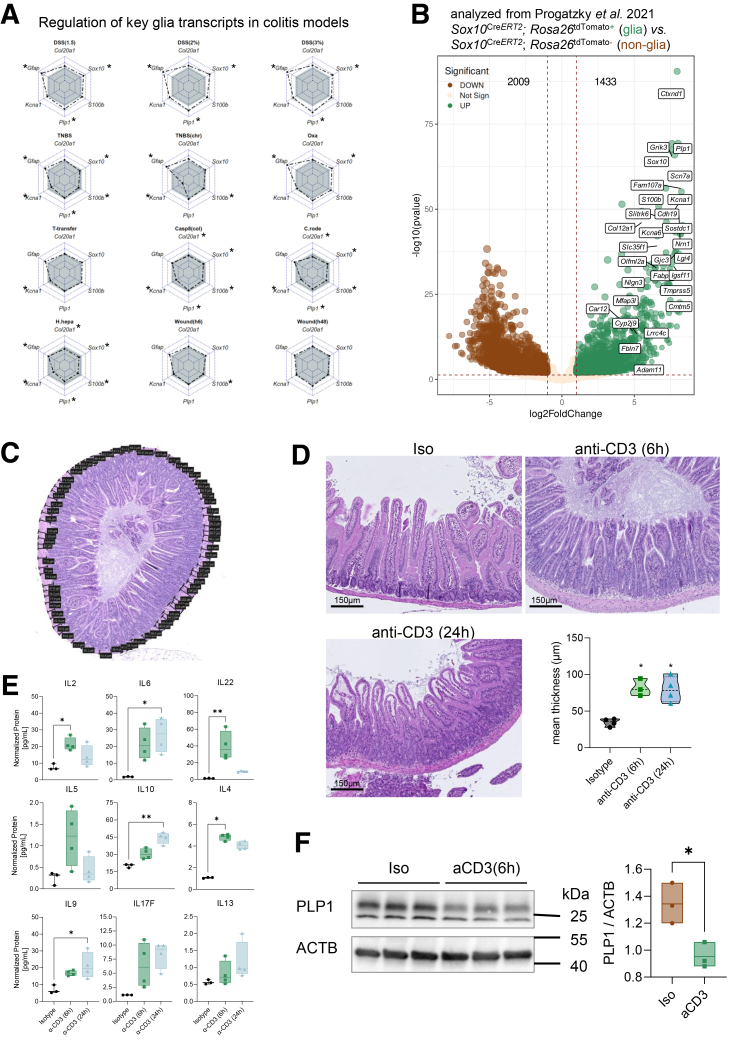
**Expression of canonical EGC markers in colitis mouse models and reduced PLP1 protein in T cell–driven gut inflammation model.** (*A*) Radar charts showing log2 fold changes of key mouse EGC genes in different paradigms of modeling colitis (Casp8(col), epithelial specific ablation of *Casp8* -induced colitis; C.rode, *Citrobacter rodentium* infection–induced colitis; DSS, dextran sulfate sodium–induced colitis; Oxa, oxazolone-induced colitis; T-transfer, adoptive T-cell transfer-induced colitis; TNBS, trinitrobenzene sulfonic acid–induced colitis; H.hepa, *Helicobacter hepaticus* infection and IL10R inhibition–induced colitis; Wound, endoscopic pinch-biopsy wound–induced colon injury. h6 and h48 indicate 6 and 48 hours post-biopsy injury).^30^^,^^34^*Asterisks* indicate *P* values <.05 from the DESeq2 Wald statistics test, *grey hexagon* outlines scale for log2 fold change at zero. (*B*) Volcano plot showing extraction of EGC-enriched markers from previously published sorted EGC vs non-EGC cells.^7^ (*C* and *D*) Detection and quantification of muscularis inflammation in the anti-CD3 antibody–induced intestinal inflammation model (aCD3). (*C*) Representative H&E image showing quantification method of muscle wall thickness from crypt base to serosa, and (*D*) comparison of muscle wall thickness in micrometers from the tissues of aCD3- vs Iso-treated mice at the indicated time points from 3 to 4 independent biological replicates per group. *Asterisks* indicate *P* < .05 from nonparametric, Mann–Whitney test. (*E*) Levels of indicated cytokines measured from duodenum of mice treated with Iso and aCD3 groups from the indicated timepoints. Data are derived from 3 to 4 biological replicates per group. *Asterisks* indicate *P* < .05 from 2-way analysis of variance followed by the Kruskal-Wallis post-hoc test. (*F*) Quantification of PLP1 protein levels of whole tissue in the indicated groups via Western blotting. *Asterisks* indicates *P* < .05 in a 2-tailed unpaired Student *t* test (n = 3/group).

The analysis of human EGC thus far hinted at the involvement of Th1-cytokine–driven pathways in the modulation of EGC in IBDs. To further investigate mechanisms underlying EGC perturbation, and to chose an appropriate experimental paradigm, we screened multiple mouse models of intestinal inflammation for EGC marker expression. Drawing from our previous evaluation of 16 IBD mouse models, we excluded several colonic inflammation protocols to avoid the complexities of dysbiosis and immune tolerance.^30^ Consistent with this, colonic models exhibited highly discordant changes in the EGC repertoire; whereas dextran sodium sulfate– and oxazolone-induced colitis were characterized by glial expansion, infectious models such as *Helicobacter hepaticus* and *Citrobacter rodentium* resulted in a marked shrinkage of the EGC population.^31^^,^^32^ Among ileitis models, anti-CD3 (aCD3) induced an upregulation of *Gfap* and repression of *Plp1*, recapitulating observations from patients with IBDs. *Eimeria vermiformis* similarly downregulated some EGC markers but only mildly induced *Gfap*. The *Casp8*_Ile model had a limited effect on EGC markers (Figures 7*A* and 8*A*).

To expand marker profiling in preclinical models, we applied the same approach used for human datasets: extracting EGC-enriched genes from sorted EGCs in inflammation (reanalyzed from Progatzky et al) (Figure 8*B*).^7^ Several genes showed rapid regulation, partially conserved with IBD datasets. As described above, given the direct T cell involvement and reduced environmental confounders, we prioritized the aCD3 model for deeper characterization. Key changes included induction of *Gfap* and *Art3*, and repression of *Plp1* (Figure 7*B*). Pathway analyses revealed repression of neuromuscular and myelination-related pathways (Figure 7*C* and *D*). These transcriptomic changes were accompanied by CD4^+^ T cell infiltration in the vicinity of enteric plexuses, increased levels of cytokines IFN-γ, TNF, and interleukin17A (IL17A) in the tissues, and muscularis inflammation (Figures 7*E, F* and 8*C–E*). These transcriptomic and cellular changes correlated with reduced PLP1 protein (Figure 8*F* and full-size blots in the Supplementary Materials) and significantly impaired small intestinal motility (Figure 7*G* and *H*).

**Figure 9:**
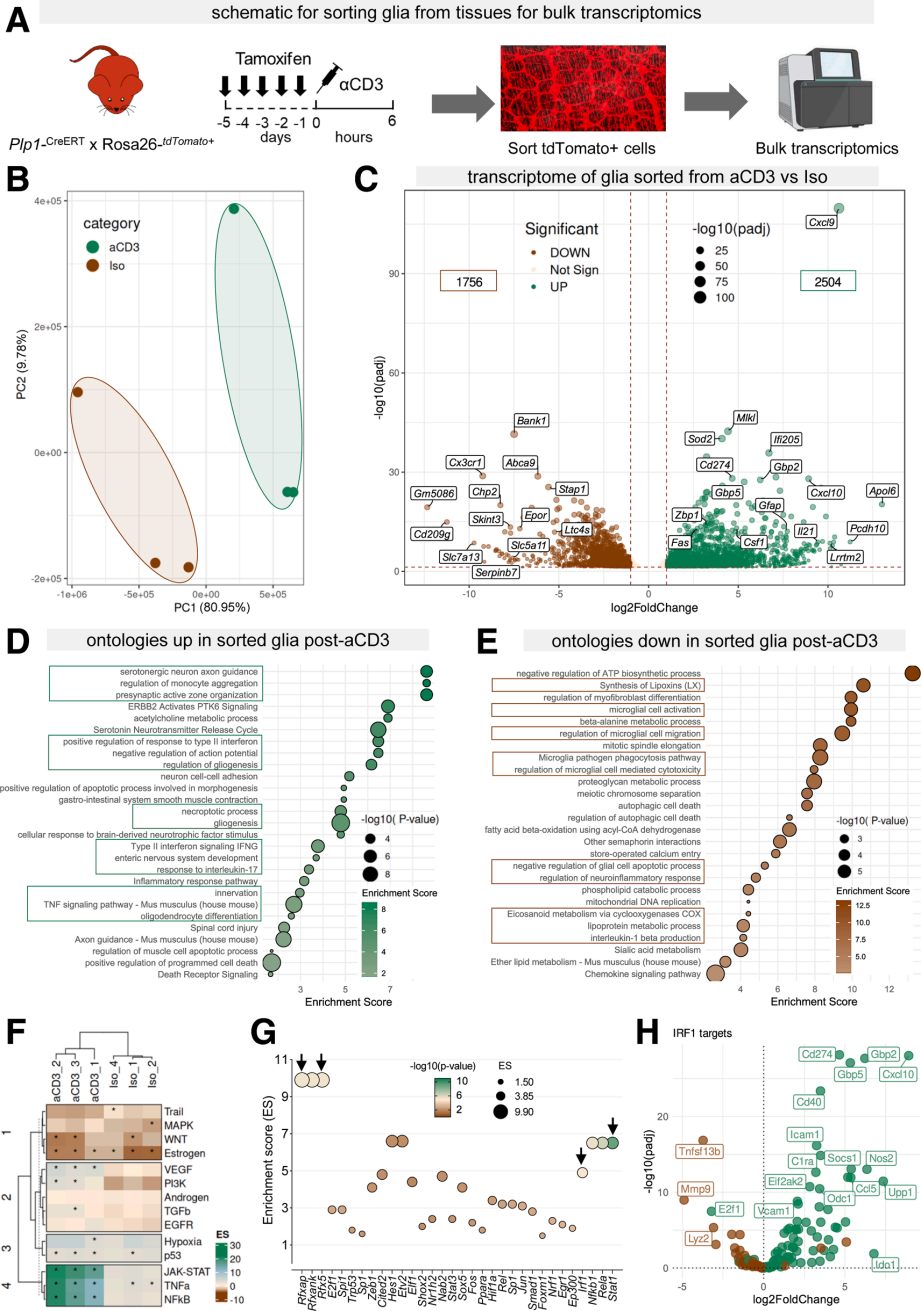
**Sorted EGC transcriptomes reveal immune-driven activation and necroptosis programs.** (*A*) Schematic of FACS-based sorting of tdTomato^+^ EGCs from *Plp1*^*CreERT+*^; Rosa26^tdTomato+^ mice following aCD3 treatment. (*B*) Principal component analysis of transcriptomes from Iso and the aCD3-treated mice. (*C*) Volcano plot showing differential gene expression (aCD3 vs Iso); *dashed lines* indicate adjusted *P* < .05 and |log_2_fold change| > .58. n = 3 per group. (*D* and *E*) Significantly enriched upregulated (*D*) and downregulated (*E*) gene ontologies; *outlined boxes* denote terms of interest. (*F*) Heatmap of pathway enrichment scores (ES) inferred via PROGENy^35^; *asterisks* denote *P* < .05. (*G* and *H*) Inferred transcription factor activity from CollectTri^36^: (*G*) Dot plot of ES and *P* values; (*H*) Volcano plot of predicted IRF1 targets, color-coded by direction of regulation.

**Figure 10:**
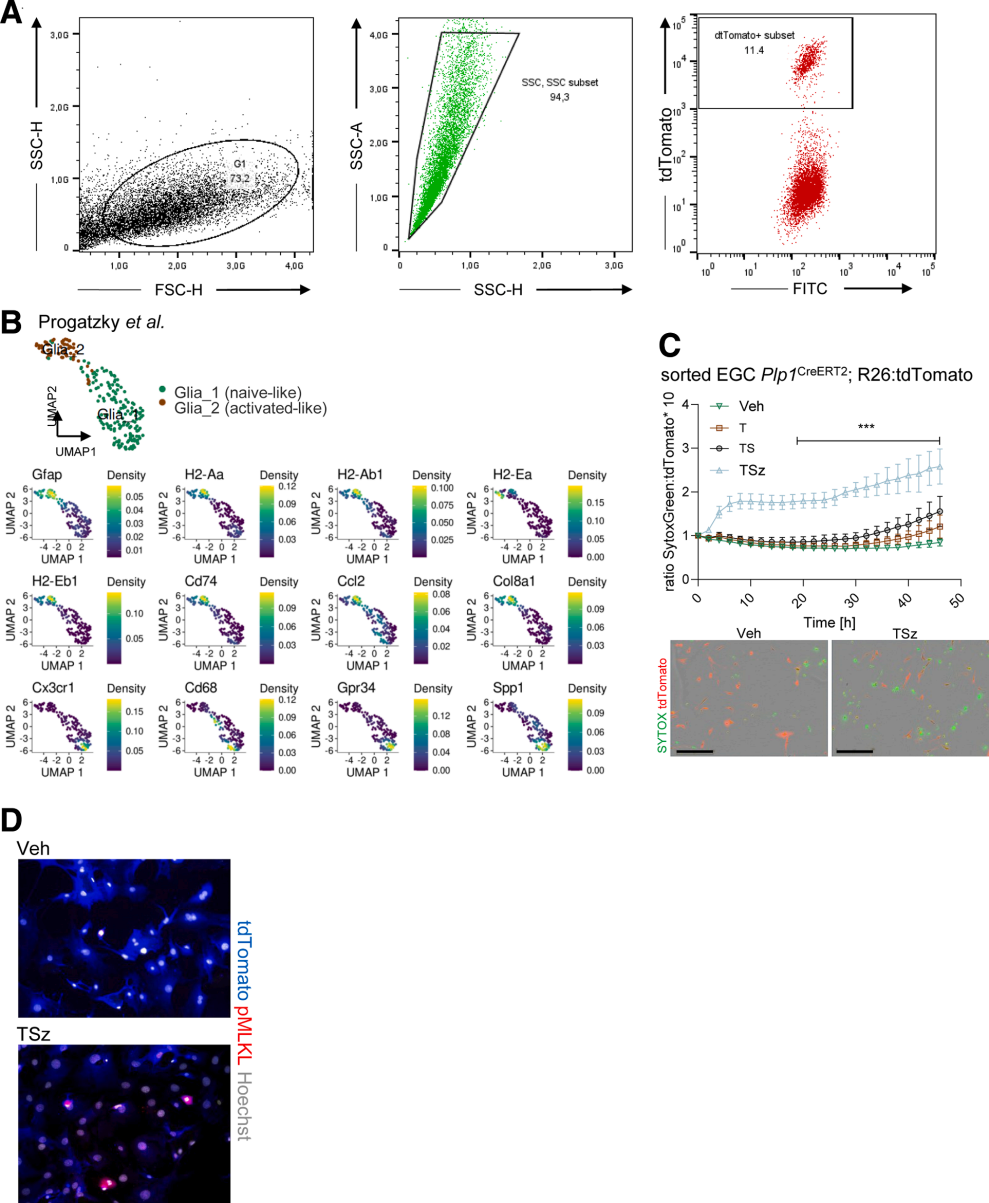
**Sorting of EGCs and EGC have the machinery to undergo necroptosis.** (*A*) Gating strategy for FACS of tdTomato+ EGC from the muscularis of glia reporter mice. (*B*) Thresholded expression densities of several activation-associated genes from the indicated clusters of mouse single EGC transcriptomes reported by Progatzky et al.^7^ (*C*) Monitoring of cell death in sorted tdTomato+ EGC from *Plp1*^CreERT2^; R26:tdTomato mice upon the induction of pharmacologic necroptosis. Veh, vehicle; T, rmTNF (50 ng.mL^−1^), TS, rmTNF (50 ng.mL^−1^) + smac mimetic LCL161 (5 μM), TSz, rmTNF (50 ng.mL^−1^) + smac mimetic LCL161 (5 μM) + pan-caspase inhibitor carbobenzoxy-valyl-alanyl-aspartyl-[O-methyl]-fluoromethylketone (zVAD-fmk) (20 μM). n = 4/group, ∗∗∗*P* < .001 2-way analysis of variance with Dunnett's multiple comparisons test. (*D*) Fluorescence micrographs of tdTomato and phospho-MLKL immunostaining for cells from the indicated conditions from the experiment shown in (*C*).

To directly assess EGC-intrinsic responses, we established the aCD3 model in *Plp1*^CreERT^; *Rosa26*^*tdTomato+*^ reporter mice, enabling tdTomato^+^ EGC sorting (Figures 9*A* and 10*A*). Transcriptomes from sorted EGCs 6 hours post-aCD3 showed marked divergence from isotype (Iso) controls, with the first principal component explaining 81% of variance (Figure 9*B*). Consistent with tissue cytokine profiles shown in Figure 7*F*, IFN-regulated genes (*Cxcl9*, *Cxcl10*), *Gfap* (7.7 log_2_ fold), and the necroptotic effector *Mlkl* (4.4 log_2_ fold) were highly induced in sorted EGC (Figure 9*C*). Ontology analysis confirmed upregulation of IFN/TNF signaling, gliogenesis, and necroptosis pathways (Figure 9*D*).

Interestingly, several downregulated genes such as *Chp2*, *Cx3cr1, Stap1,* and *Epor* hinted at repressed adaptive immune and stress responses (Figure 9*C*). The inverse regulation of *Gfap* and *Cx3cr1* aligned with previous single-cell RNA sequencing (scRNA-seq) findings, also showing differential EGC activation and immune gene expression (Figure 10*B*).^7^ Altogether, several downregulated ontologies were related with microglia activation and regulation of neuroinflammatory and stress response genes (Figure 9*E*). Correspondingly, these downregulated ontologies involved microglial activation, neuroinflammation, and stress response (Figure 9*E*). Pathway and transcription factor activity inference confirmed TNF, Janus kinase (JAK)–signal transducer and activator of transcription (STAT), and nuclear factor κB as key upstream pathways and STAT1, IRF1, and RFX family TFs as major regulators (Figure 9*F–H*).^36^^,^^37^ High HES1 activity, a known regulator of ENS development, was also observed (Figure 9*G*).

**Figure 11:**
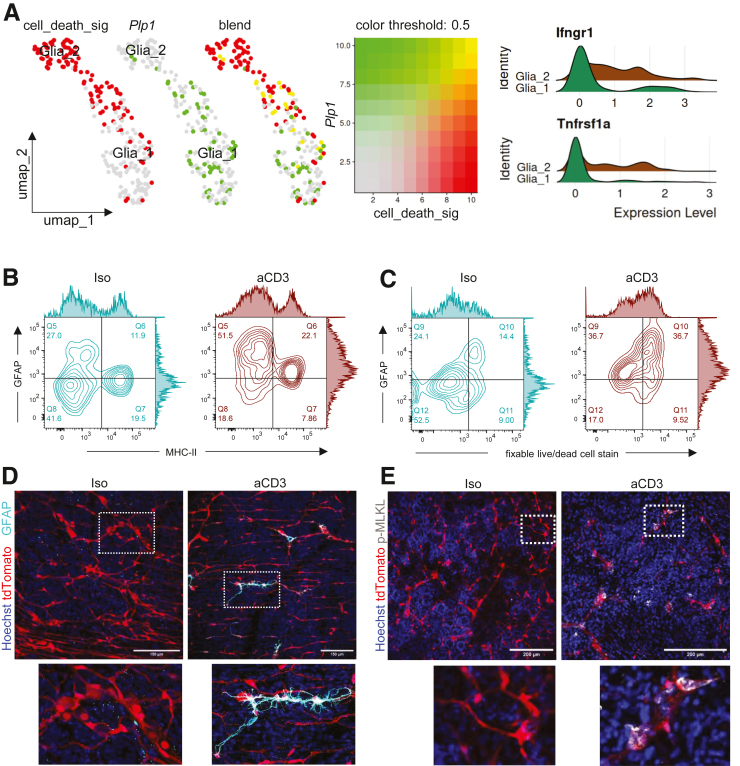
**Necroptosis induction in activated EGCs during Th1/Th17-driven intestinal inflammation.** (*A*) Uniform Manifold Approximation and Projection (UMAP) plots reanalyzed from Progatzky et al 7 showing EGC clusters with elevated cell death signature and reduced *Plp1* expression. Ridge plots display *Ifngr1* and *Tnfrsf1a* expression in Glia_2 cluster with higher expression of the cell death pathway signature and lower *Plp1* expression. (*B* and *C*) Flow cytometry density plots of tdTomato^+^ EGCs from aCD3- or Iso-treated mice, showing activation markers and cell death dye. (*D* and *E*) Whole-mount images of submucosal plexus: (*D*) GFAP and tdTomato colocalization, (*E*) pMLKL staining in tdTomato^+^ EGCs from aCD3 vs Iso groups.

**Figure 12:**
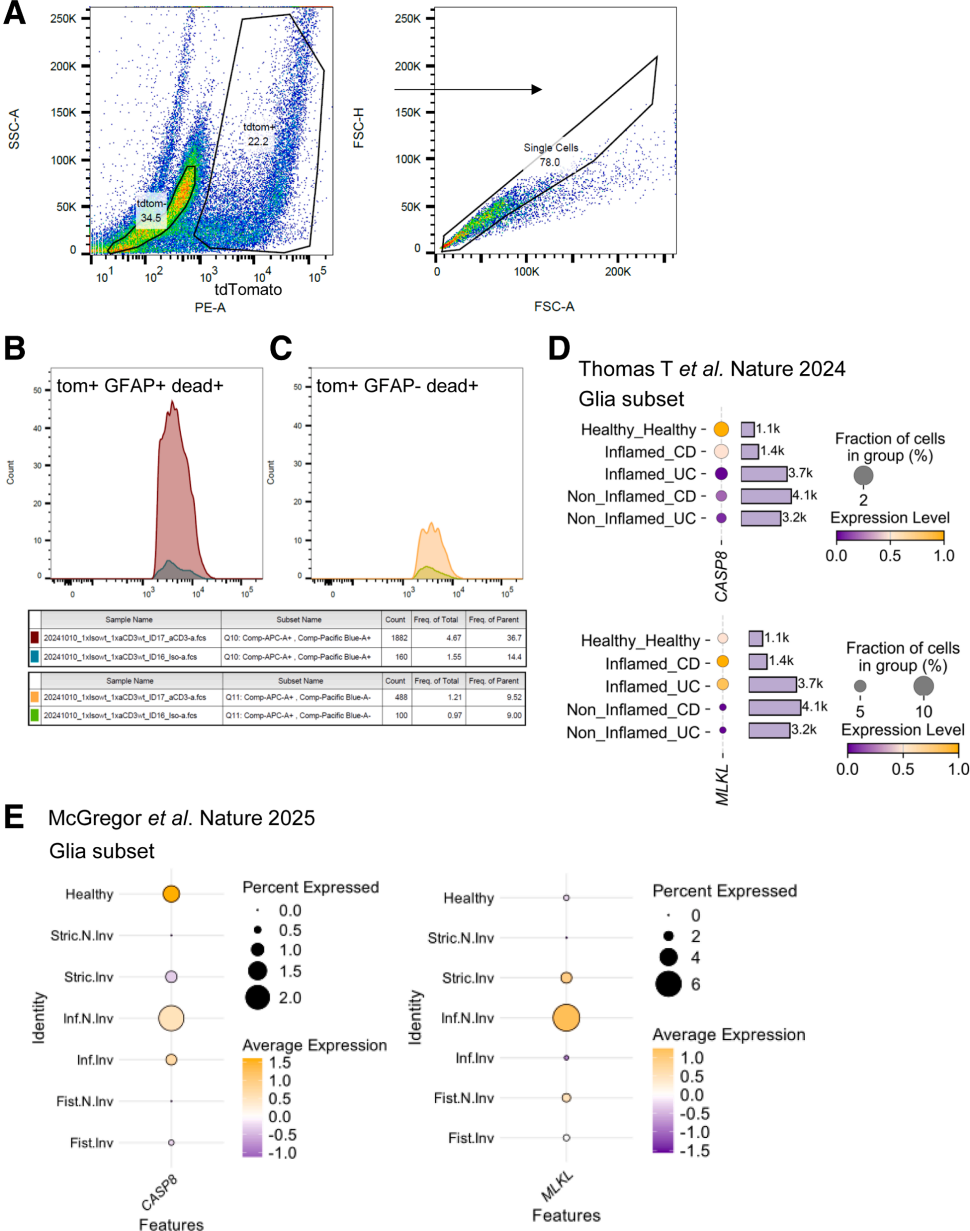
**Induction of cell death in activated EGCs in vivo.** (*A*) Plots showing gating strategy for tdTomato+ single EGC from digested muscularis samples for further analysis. (*B* and *C*) Activated (tdTomato+, GFAP+) live (*B*) and dead (fixable dead cell dye+) (*C*) EGC and their proportions in aCD3 and Iso groups. (*D* and *E*) Analysis of glia subsets from 2 independent scRNA-seq datasets^29^^,^^38^ showing the expression of *CASP8* and *MLKL* in the indicated patient groups. Fist.Inv, fistulating involved; Fist.N.Inv, fistulating noninvolved; Inf.Inv, inflammatory involved; Inf.N.Inv, inflammatory noninvolved; Stric.N.Inv, stricturing noninvolved; Stric.Inv, stricturing involved.^38^

Given the induction in the IFN-TNF and necroptosis ontologies, we directly tested whether EGCs could execute cellular necroptosis. Sort-purified EGCs were susceptible to conventional pharmacologically induced necroptosis with an elevation in phospho–mixed lineage kinase domain-like pseudokinase (MLKL) immunopositivity (Figure 10*C* and *D*). Apoptosis could be ruled out as carbobenzoxy-valyl-alanyl-aspartyl-[O-methyl]-fluoromethylketone failed to rescue from such cell death. An elevation of the cell death signature along with elevated expression of the *Ifngr1* and *Tnfrsf1a* receptors in the activated EGCs was also observed on the dataset by Progatzky et al,^7^ mirroring our sorted EGC data (Figure 11*A*; Table 1). We next assessed whether such EGC necroptosis accompanied with activation occurs in vivo. EGC activation is typically marked by GFAP and MHC-II upregulation.^6^ Flow cytometry of tdTomato^+^ EGCs post-aCD3 showed a 2-fold increase in GFAP^+^ MHC-II^+^ cells and a 2.5-fold rise in GFAP^+^ cells stained with viability dye compared with Iso controls (Figures 11*B, C* and 12*A–C*). Moreover, whole mount immunostaining showed higher GFAP+ tdTomato+ EGC in the submucosal plexuses of aCD3-, but not Iso-treated mice (Figure 11*D*). Interestingly, immunopositivity of the necroptosis executioner phospho-Ser345 MLKL (pMLKL) was also higher in the aCD3- vs Iso-treated group (Figure 11*E*), confirming EGC necroptosis also occurs in vivo. To validate necroptosis in human IBD EGCs, we analyzed *CASP8* and *MLKL* expression across 2 independent scRNA-seq datasets.^29^^,^^38^ Inflamed IBD EGCs exhibited elevated *MLKL* and reduced *CASP8* levels, typical for a pivot towards necroptosis (Figure 12*D* and *E*). Collectively, these data indicate that EGC activation and MLKL-dependent necroptosis can be regulated via TNF and IFN-γ signaling, a signature confirmed in inflamed human IBD datasets.

### Interferon-γ and Tumor Necrosis Factor Drive Activation and Necroptosis Induction in Sorted Enteric Glial Cells Ex Vivo

**Figure 13:**
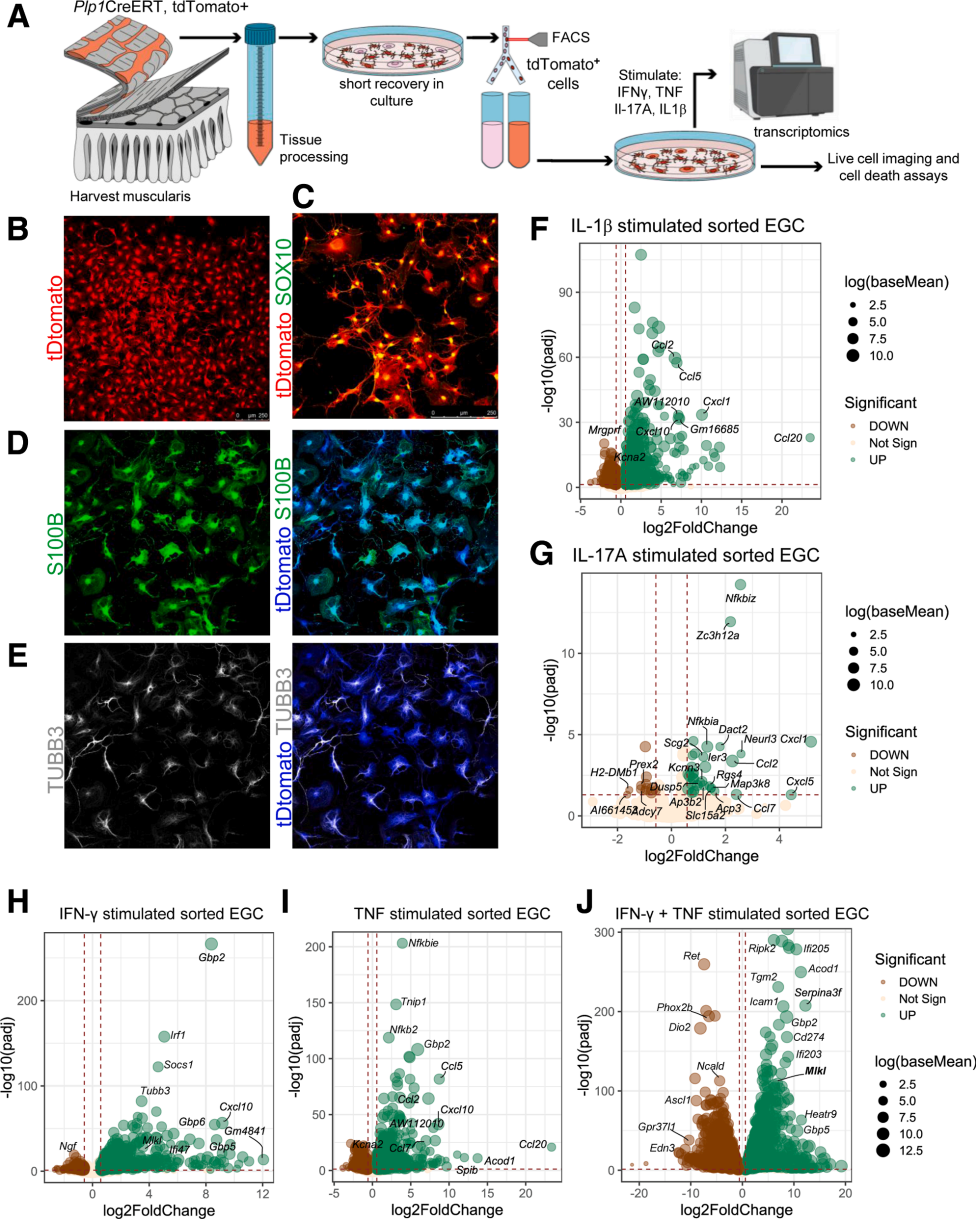
**Coordinated action of IFN-γ and TNF drive EGC activation, necroptosis, and repress enteric gliogenesis.** (*A*) Schematic depiction of the protocol for the establishment of FACS sorting tdTomato+ EGC and culturing them ex vivo for transcriptomic and live cell imaging evaluation. (*B*) Representative photomicrographs of sorted EGCs showing tdTomato reporter expression, traceable in live culture. (*C–E*) Representative photomicrographs from sorted EGCs, fixed and immunostained for the canonical EGC marker SOX10 (*C*) and S100B (*D*) showing that all sorted tdTomato+ cells are positive for SOX10 and S100B with some expression of the neuronal marker TUBB3 (*E*) after 10 days in ex vivo culture. (*F–J*) Volcano plots showing transcriptomic responses of sorted EGCs to IL1β (*F*), IL17A (*G*), IFN-γ (*H*), TNF (*I*), and IFN-γ + TNF (*J*) stimulations followed by bulk RNA-seq ex vivo. Data is derived from n = 3–4/group biological replicates. *Dashed brown line* along the x-axis denotes adjusted *P* value threshold of .05 for the DESeq2 Wald statistics test. *Vertical dashed brown lines* indicate absolute log2 fold change threshold of 0.58.

**Figure 14:**
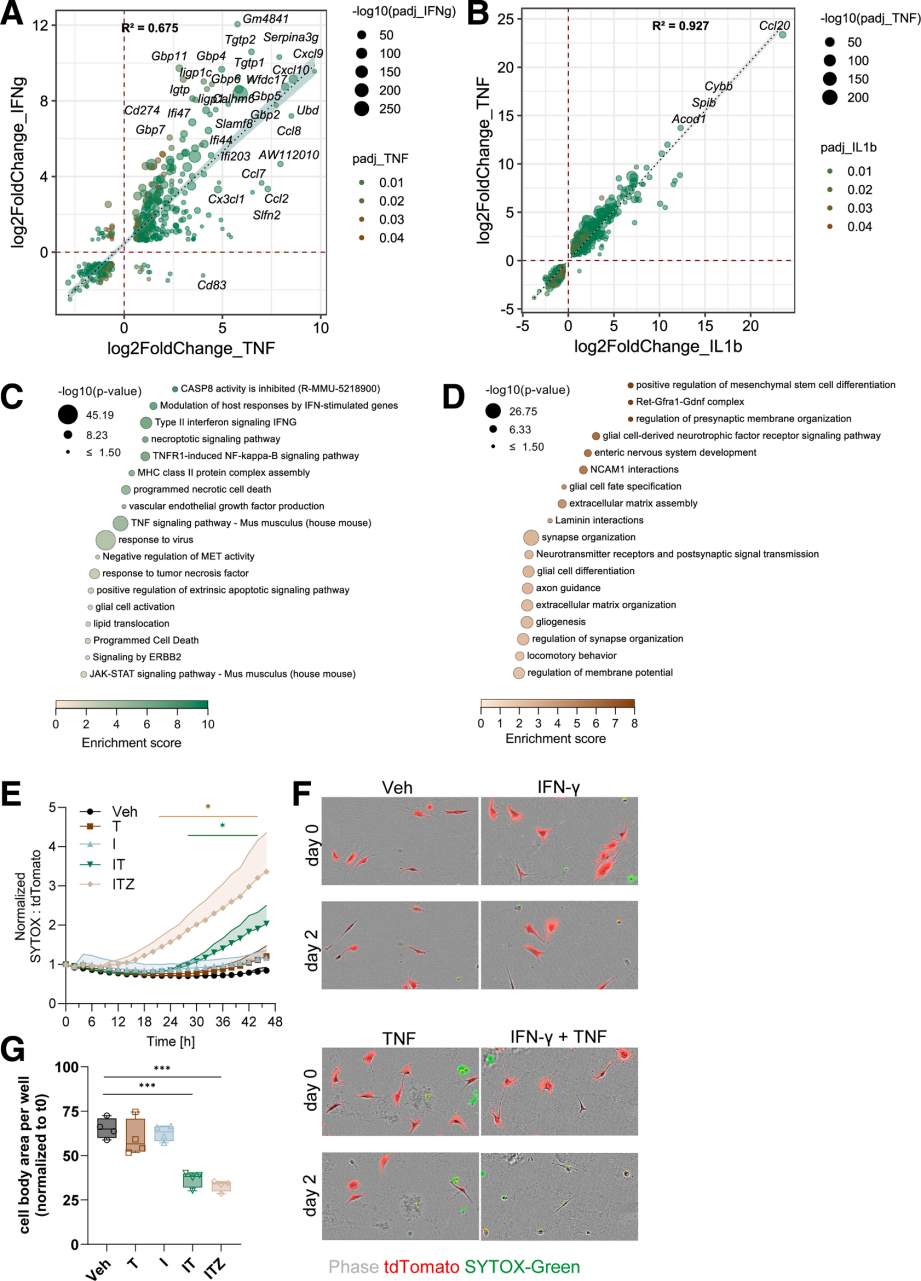
**IFN-γ and TNF drive necroptosis and repress enteric gliogenesis.** (*A* and *B*) Scatter plots showing transcriptome-transcriptome comparison of shared genes between TNF vs IFN-γ (*A*) stimulated and IL1β vs TNF (*B*) stimulated sorted tdTomato+ EGCs. (*C* and *D*) Selected upregulated (*C*) and downregulated (*D*) ontologies from the transcriptomes of sorted EGCs costimulated with IFN-γ and TNF. (*E*) Live-cell tracking of tdTomato^+^ EGCs over 48 hours following cytokine stimulations ± carbobenzoxy-valyl-alanyl-aspartyl-[O-methyl]-fluoromethylketone (zVAD-fmk). Veh, vehicle; T, TNF; I, IFN-γ; Z, zVAD-fmk. (*F*) Representative images from the tdTomato+ EGCs and Sytox-Green from the last time point of the experiment shown in (*E*). (*G*) Quantification of cell area coverage at the end of the experiment, normalized to time 0 hours for the experiment shown in (*E*). For (E–G), n = 4/group. In (O), ∗*P* < .05 in 2-way analysis of variance with Holm–Šidák’s test, in (Q), ∗∗∗*P* < .001 in 1-way analysis of variance with Tukey’s test.

To directly test the effects of Th1/Th17 cytokines on EGC activation and death, we optimized ex vivo culture of fluorescence-activated cell sorting (FACS)-sorted EGCs from *Plp1*^*CreERT+*^; *Rosa26*^*tdTomato+*^ mice (Figure 13*A*). Cultured tdTomato^+^ cells retained expression of canonical EGC markers SRY-box transcription factor 10 (SOX10) and S100B, and partially of TUBB3 after 10 days (Figure 13*B–E*).^39^ We stimulated these EGCs with specific IBD-associated cytokines: interleukin1β (IL1β), IL17A, IFN-γ, and TNF.^7^^,^^26^^,^^40^^,^^41^ Analysis revealed that IFN-γ, TNF, and IL-1β elicited strong transcriptomic responses, whereas IL-17A had a more modest impact (Figure 13*F–J*). Both IFN-γ and TNF induced inflammatory genes *Cxcl9* and *Ccl2*, similar to the inflamed human EGC signature found from sn- and sc-RNA-seq datasets (Figure 14*A*). There was also a very high degree of transcriptomic overlap (R^2^ = 0.927) between the IL1β- and TNF-stimulated EGC, mostly owing to the autocrine TNF expression upon IL1β stimulation (Figure 14*B*).

Reflecting the coelevation of IFN-γ and TNF in aCD3 tissues shown in Figure 7*F*, we identified a synergistic effect where costimulation drove robust upregulation of *Mlkl*, *Cd274*, *Gbp2*, and *Ifi205*, mimicking the profile of aCD3-sorted EGCs (Figures 9*C* and 13*J*). Gene ontology analysis showed enrichment of necroptotic signatures, including inhibition of caspase-8 activity and induction of programmed necrosis (Figure 14*C*), consistent with known roles for caspase-8 in necroptosis regulation.^42^ Moreover, costimulation also repressed *Ret*, *Phox2b*, *Gpr37l1*, *Edn3*, and *Ascl1*, genes vital to ENS homeostasis (Figure 13*J*). This was reflected in significant enrichment in selected downregulated gene ontology terms related with glial cell and ENS differentiation (Figure 14*D*).

**Figure 15:**
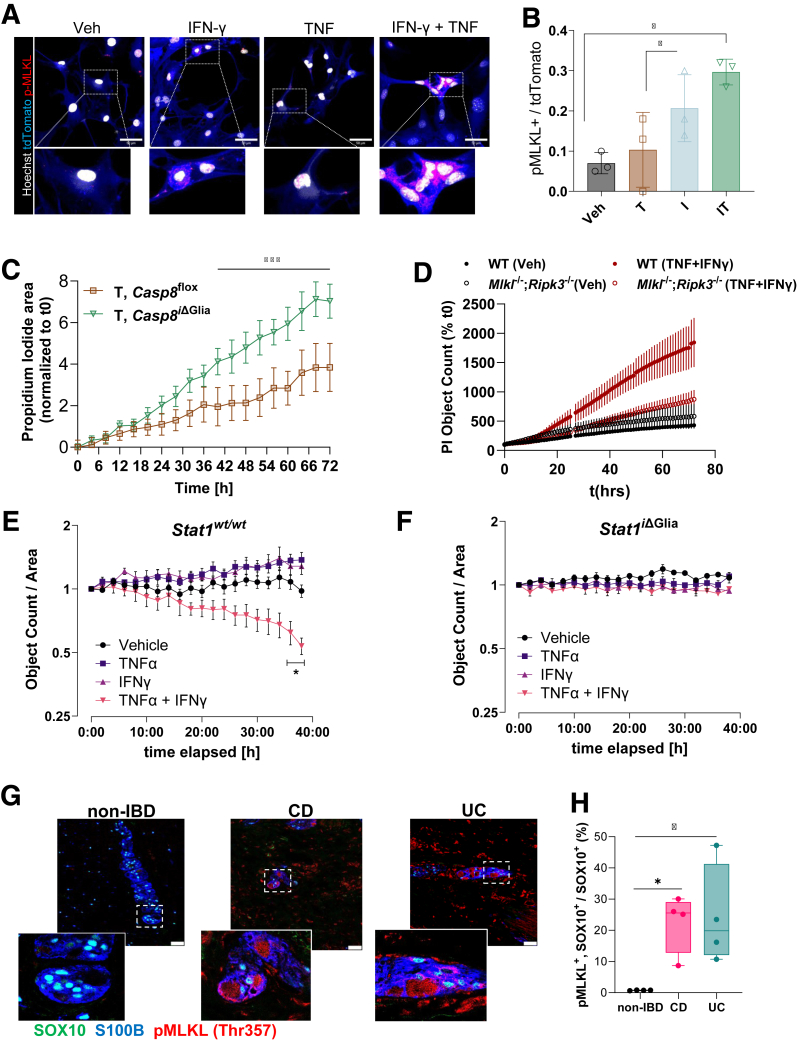
**IFN-γ and TNF coordinate EGC death enhanced by *Casp8* ablation.** (*A* and *B*) pMLKL immunostaining of EGCs after 48-hour stimulation, with pMLKL/tdTomato intensity quantified (n = 3); *P* < .05 by analysis of variance with Tukey’s post-hoc test. (*C*) Propidium iodide–based death tracking in plexanoids from glia-specific *Casp8* knockout (*Casp8*^*iΔGlia*^) or CTRL mice (*Casp8*^*flox*^), pre-treated with 4-hydroxytamoxifen and stimulated with TNF. n = 3/group, ∗∗∗*P* < .001 in 2-way analysis of variance with Tukey’s test. (*D*) Propidium iodide–based death tracking in plexanoids from wild-type (WT) or *Mlkl−/− Ripk3−/−* double deficient mice stimulated with vehicle (Veh) or TNF+ IFN-γ. n = 2/group, average of experimental replicates is plotted. (*E* and *F*) Live cell imaging of tdTomato+ sorted EGCs from *Stat1*^wt/wt^ control (*E*) or *Stat1*^iΔGlia^ (*F*). EGC stimulated with the indicated cytokines and combinations. Both genotypes were on a R26:tdTomato+ background and both received tamoxifen at 75 mg.kg^−1^ for 5 days on/2 days off cycle thrice before FACS sorting and culturing. The *P* < .05, in 2-way analysis of variance with Dunnet’s multiple comparisons test. n = 3/group. (*G* and *H*) Immunostained resection tissues revealing SOX10+, S100B+, pMLKL+ EGCs in patients with IBD (*G*) and the enumeration of these vs total EGCs (*H*). *P* < .05 in a nonparametric Mann–Whitney test, n = 4/group.

These data suggest that IFN-γ and TNF jointly drive EGC activation and necroptosis pathway induction. To assess functional outcomes, we performed live imaging of tdTomato^+^ EGCs stimulated with IFN-γ, TNF, or both. Neither cytokine alone caused any appreciable cell death, but costimulation led to marked increase in cell death, causing a reduced cell area (Figure 14*E–G*). Caspase blockade with carbobenzoxy-valyl-alanyl-aspartyl-[O-methyl]-fluoromethylketone failed to rescue from this IFN-γ + TNF–induced cell death; instead, it exacerbated it, consistent with regulated necrosis (Figure 14*E–G*).^43^ Moreover, a significant elevation in pMLKL immunopositivity and an increase in the pMLKL to tdTomato signal intensity was detectable in sorted EGCs costimulated with IFN-γ and TNF (Figure 15*A* and *B*).

**Figure 16:**
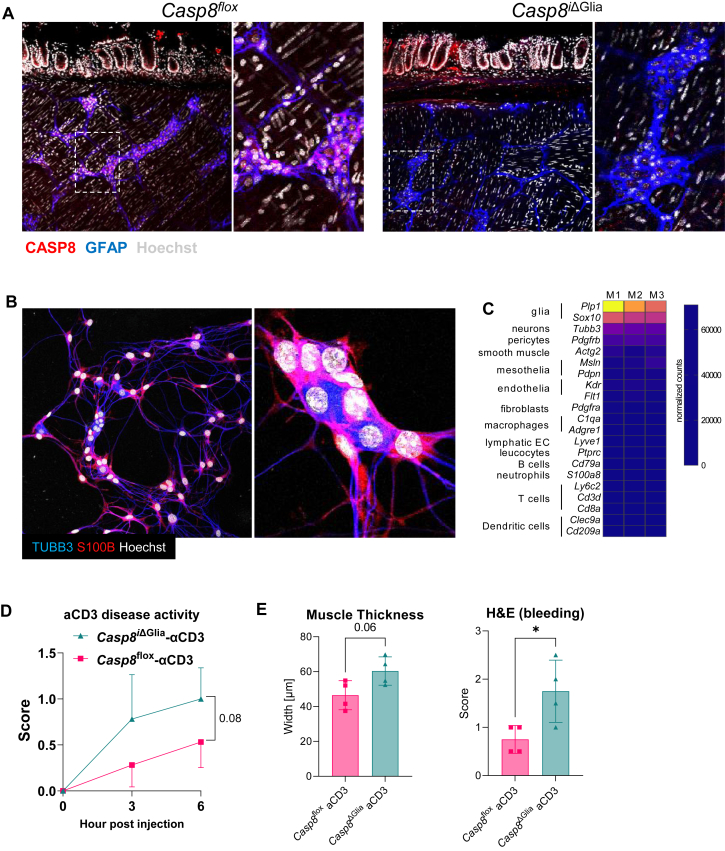
**Glia-specific Casp8 deficiency increases susceptibility to T cell–driven enteritis.** (*A*) Whole mount immunostaining for CASP8 and GFAP from the colon tissues of *Casp8*flox and *Casp8*iΔGlia mice on day 10 post 3 cycles of tamoxifen administration, showing the glial specificity of the knockout. *Insets* show magnified views of selected areas. (*B*) Representative photomicrographs of immunostained plexanoids cultured from intestines of C57BL/6J mice displaying S100B-positive EGCs surrounding TUBB3-positive neurons, displaying morphologies reminiscent of plexuses in culture. (*C*) Heatmap visualizing normalized counts of selected cell type marker genes from bulk RNA-seq delineating the composition of plexanoid cultures from 3 independent biological replicates (M, mouse: M1 to M3) showing dominance of EGCs in the culture. (*D* and *E*) Mean with standard deviation of disease activity scores (*D*), muscle thickness and H&E bleeding scores (*E*) from littermate *Casp8*flox controls and *Casp8* glia-deficient *Casp8i*ΔGlia mice, treated with 75 mg per kg body weight of tamoxifen for 5 days, followed by the induction of intestinal inflammation via the aCD3 model. Data are derived from 4 independent biological replicates. *Asterisks* in (*E*) indicate *P* < .05 in Welch’s 2 tailed *t* test.

Given the transcriptomic and functional evidence for caspase-8 inhibition, we hypothesized that glial *Casp8* deletion would sensitize EGCs to TNF. We generated *Plp1*^*CreERT+*^*; Casp8*^*flox/flox*^ mice (*Casp8*^*iΔGlia*^) to conditionally and inducibly delete *Casp8* in EGCs. Induction of the knockout via tamoxifen led to a glia-specific loss of caspase-8 protein, shown by whole mount immunostaining (Figure 16*A*). Caspase-8 is a key regulator of TNF-mediated apoptosis, and its loss predisposes several cell types to necroptosis.^42^^,^^44^^,^^45^ Time-lapse imaging of plexanoids showed that TNF alone was enough to trigger significant cell death in *Casp8*^*iΔGlia*^ but not in CTRL (*Casp8*^*flox*^) EGCs (Figure 15*C*) and plexanoids (Figure 16*B* and *C*). In line with elevated necroptosis already observed in the aCD3 model in wild-type mice, the aCD3-induced muscularis inflammation, disease scores, and muscle thickness remained comparable between *Casp8*^*i*ΔGlia^ and *Casp8*^*flox*^ mice. However, *Casp8*^*iΔGlia*^ mice showed increased intraluminal and parenchymal red blood cells (Figure 16*D* and *E*), consistent with enhanced barrier disruption or vascular leakage.

To conclusively prove the involvement of the necroptosis cascade in EGC via IFN-γ + TNF, we next showed a rescue from cell death in plexanoid cultures derived from mice doubly deficient in *Mlkl* and *Ripk3* (Figure 15*D*). Co-operativity between IFN-γ and TNF in inducing multiple parallel modes of cell death has been established under certain conditions such as autoimmunity and viral infections. These include the newly discovered “panoptosis”^46^ and a CASP8-JAK1/2-STAT1–dependent cell death.^47^ Having shown a clear induction in necroptosis coupled with the increased expression of *Mlkl* (Figures 9*C* and 13*J*) and the involvement of STAT1 in regulating the transcriptome of the sorted EGCs in the aCD3 model (Figure 9*G*), we hypothesized that the STAT1 arm may also play a role in regulating EGC necroptosis. For this, we generated mice with conditional and inducible glia-specific *Stat1* ablation on the tdTomato background (*Stat1*^iΔGlia^). Sort-purified tdTomato+ EGCs from *Stat1*^iΔGlia^ were also rescued from the TNF+IFN-γ–induced cell death compared with the *Stat1*^fl/fl^ controls (Figure 15*E* and *F*). These findings highlight that TNF and IFN-γ cooperate to induce MLKL- and STAT1-dependent cell death in EGCs. Taken together, these data identify IFN-γ and TNF as cooperative inducers of regulated necrosis in activated EGCs.

Finally, to identify the translational relevance of our findings, we performed a triple-immunolabeling in resection tissue sections from our IBDome patient cohort to identify SOX10+, S100B+ EGCs that are pMLKL+ necroptotic. We saw a significant elevation in necroptotic EGCs in both CD and UC compared against non-IBD resection tissues, directly revealing EGC necroptosis in IBDs (Figure 15*G* and *H*).

Our findings identify EGC activation and necroptosis as key components of glial adaptation to inflammation, with coordinated TNF–IFN-γ signaling contributing to necroptosis in clinical and preclinical IBDs. However, given the limited involvement of IFN-γ in UC,^48^^,^^49^ necroptosis in this context likely depends on TNF in combination with additional, as yet incompletely defined, inflammatory mediators.

## Discussion

The precise roles and the impact of inflammation on EGCs in IBDs are under-recognized. Traditionally considered neuroprotective and neurotrophic, the immune and epithelial regulatory roles of activated EGCs in inflamed guts are just beginning to be uncovered.^50^ Enteric and peripheral neuropathy is frequently observed in IBDs, although the exact origins remain elusive, with speculation that the path to immune attack on neuroglia might be paved first via dysfunctional immune–EGC crosstalk. There is growing evidence from mouse models suggesting that, specifically, the *Gfap* expressing, activated EGCs exert several beneficial functions. This includes activated EGC-derived Wnt in improved epithelial recuperation postinjury as well as improved helminth clearance.^3^^,^^51^ Moreover, in mice, activated EGCs can induce tolerogenic T cells via MHC-II–mediated intrinsic antigen presentation.^6^ Despite these reports, recent single-cell studies have cast some doubt on the specificity of GFAP in labeling EGCs.^8^^,^^9^^,^^52^ Therefore, there is an emergent need for the identification of EGC-specific markers that are also inflammation-associated. Our study addressed this need by extracting EGC-enriched genes from an integrated collection of IBD sc transcriptomes. This led us to some interesting and unexpected EGC-enriched genes, with as yet unknown implications in immune and epithelial crosstalk. For instance, we found that EGCs from inflamed IBD samples displayed higher expression of *SPP1* on multiple datasets from colon tissues of IBDs. Previous reports indicate that in mice, small intestinal intraepithelial lymphocytes can express *SPP1*. However, its dominant cellular source in the human colon has not been defined. Given that SPP1 is a secreted protein and showed a robust correlation with histologic severity in our cohorts, it could potentially serve as a noninvasive surrogate marker for monitoring glial network integrity and disease progression via serum or stool assays. Furthermore, the glial death signature identified in our study could provide a basis for patient stratification. Patients exhibiting a high activated–lethal glial profile at the time of diagnosis might represent a subgroup at higher risk for motility-related complications, potentially requiring earlier escalation to advanced biological therapies.

Another important function of EGCs, which has become increasingly appreciated in recent years, is their ability to carry out injury-induced neurogenesis, replenishing lost ENS cells in mouse models.^53^^,^^54^ This is enabled due to the existence of a multipotent PLP1 expressing glia-like quiescent cells in the myenteric plexus. Data from patients with IBDs has so far not reported the presence of such a population. One of the EGC subclusters identified on our snRNA-seq is a *PLP1*-high cluster, which simultaneously coexpressed a number of neural crest precursor genes, including *PHOX2B*, *SOX2*, and *RET*. Moreover, this cluster was rich in patient EGCs compared with those from non-IBD CTRLs, indicating that inflammation may trigger the emergence of neurogenic EGCs in IBDs. Ours is the first report to describe such a cluster, presumably given that we analyzed sn from full-thickness gut tissues, avoiding the cell health–related losses that affect single-cell isolation protocols. Interestingly, this methodology also provides insight into the spatial heterogeneity of glial activation. Although activation markers are often more apparent in mucosal or submucosal biopsies, our data suggest that glial activation is most pronounced in these relatively superficial layers. This spatial gradient may explain the apparent lack of marker activation in patients with CD despite high IFN-γ and TNF exposure. Because CD is a transmural disease, full-thickness resection samples contain a higher proportion of myenteric glia. These deeper populations may be less exposed to the mucosal inflammatory milieu compared with the glia captured in UC mucosal biopsies. However, our data from the full-thickness snRNA-seq did identify that, even in CD, the EGCs had an overall induction in cell death signature, albeit slightly lesser than that observed in the activated UC EGC clusters.

Pathologic, caspase-independent TNF- and IFN-γ–driven cell death has been previously reported in brain oligodendrocytes.^55^ However, ours is the first report on extrinsic necroptosis induction in response to inflammatory cytokines in EGCs. This is particularly relevant in IBDs, where immune cell infiltration into ENS plexuses alters neuroglial function via local cytokine production,^56^ but its impact on EGC health has been largely ignored. Elevation of TNF and IFN-γ in tissues is known to synergistically coordinate inflammatory necroptosis via heavily inducing expression and phosphorylation of the necroptosis executioner MLKL.^57^ Other gastrointestinal cell types have also been shown to undergo necroptosis in response to IFN-induced MLKL upregulation, and our findings extend this paradigm to EGCs.^44^^,^^58^ Furthermore, our data indicate that IFN-γ and TNF predispose EGCs to necroptosis in a manner that involves MLKL and is dependent on STAT1. Recent evidence from intestinal epithelial cells indicates that STAT1, partly via transcriptional upregulation of *Mlkl*, can regulate necroptosis sensitivity.^59^ However, unlike the intestinal epithelia, where IFN-γ and TNF synergize to induce panoptosis^57^ and caspase8-JAK1/2-STAT1^47^–mediated cell death, we show that EGCs undergo an MLKL- and STAT1-dependent necroptosis in response to IFN-γ and TNF. Further investigation is required on the control of EGC transcriptome via STAT1.

However, it is intriguing that pMLKL levels were comparably elevated in both CD and UC tissues, although there is limited involvement of IFN-γ in UC. Therefore, it is important to note that our data identify 2 related but mechanistically distinct glial responses during intestinal inflammation: (1) enteric glial activation; and (2) glial necroptotic cell death. Although glial activation is particularly prominent in superficial regions of UC tissue, we do not interpret this phenotype as primarily driven by IFN-γ signaling. The contribution of IFN-γ in our study is more closely linked to the induction of necroptosis, where cooperative TNF–IFN-γ signaling has been well described in inflammatory contexts and is consistent with the cytokine milieu observed in CD. However, given the limited evidence for IFN-γ at the protein level in UC,^48^^,^^49^ it is unlikely that this axis alone accounts for the glial necroptosis observed in UC.

Instead, our findings hint at a model in which TNF cooperates with additional inflammatory inputs present in the UC mucosa. Although IFN-associated transcriptional signatures are frequently reported in UC, direct evidence for corresponding protein-level changes remains limited. IFN pathways beyond IFN-γ may nonetheless be relevant, as type III IFNs have been implicated in the regulation of necroptosis in intestinal epithelial cells (for example, through modulation of MLKL expression).^60^ Type III IFNs such as IFN-λ therefore may represent a plausible, albeit not yet definitively established, component of the UC glia necroptosis phenotype. In parallel, other factors characteristic of inflamed mucosa may contribute to lowering the threshold for necroptotic cell death, including alternative death receptor ligands such as FasL or TRAIL, microbial-derived signals engaging pattern recognition receptors in the context of barrier disruption, and local metabolic or oxidative stress conditions. Collectively, these signals may act in concert with TNF to lower the necessary threshold for necroptosis in UC enteric glia. Thus, rather than a single dominant cytokine driver, glial cell death in UC likely reflects the integration of multiple proinflammatory cues within a complex and as yet incompletely defined mucosal environment.

Overall, our study provides new insights into the inflammatory regulation of EGC turnover in intestinal inflammation. Moreover, our data indicate that small-molecule inhibitors of RIPK3 or MLKL, several of which are currently in clinical trials for other inflammatory conditions, that target the necroptotic pathway, may offer a novel neuroprotective, gut gliovascular barrier–protecting, and motility–promoting strategy in IBDs.

A differential impact of inflammation on EGC subtypes and the functional consequences to gut physiology in IBDs remains an open question. It is likely that EGC turnover is influenced in patients who respond to anti-TNF therapy via reduced activation and associated cell death. Given that EGC subsets regulate gut motility, it is interesting to note that previous and ongoing clinical trials in CD have proposed that magnetic resonance imaging–assisted monitoring of gastrointestinal motility might be a predictor of anti-TNF therapy response.^61^ The protection from cytokine-induced EGC death may extend to EGC clusters with different functions. Further studies aimed at understanding the molecular and functional diversity of EGC subtypes and their implications in IBDs will provide an improved understanding of the complex crosstalk between the enteric nervous system’s role in maintaining neuro–immune–epithelial homeostasis in the gut.

## Materials and Methods

### Human Cohorts

For the in-house IBDome cohort, samples were obtained from patients with and without IBDs with written informed consent under ethics approvals EA1/200,717 (Berlin) and 332-17B (Erlangen). Further details of the cohort and data are accessible at https://ibdome.org/#!/.^62^ A breakdown of the cohort used in this study, including demographics (age at sampling/sex), disease subtype (UC vs CD), tissue, and sampling method is provided in Table 2. Resected tissue and biopsies were collected during endoscopy at Charité – Universitätsmedizin Berlin and Universitätsklinikum Erlangen (Gastroenterology and Surgery Departments). Samples were stored overnight in 10 mL RNAprotect Tissue Reagent (Qiagen) at 4 °C; after removal of the reagent, biopsies were stored at −80 °C. RNA was isolated using the RNeasy Kit (Qiagen) with the TissueLyser LT and 5-mm steel beads. RNA concentration and purity (A260/280, A260/230) were measured with a Nanodrop 1000, and RNA integrity number assessed using the Agilent 2200 TapeStation (RNA ScreenTape System). Low–RNA integrity number samples were processed with the RNA Clean & Concentrator Kit. Frozen samples were shipped on dry ice to the Quantitative Biology Center (QBIC), Tübingen, for sequencing. All patient data were pseudonymized. The final cohort included CD (n = 277), UC (n = 121), and non-IBD (n = 46) samples (196 male, 198 female). Replication analyses used public bulk and scRNA-seq datasets: Kinchen et al (GSE114374)^22^; Nie et al (http://scibd.cn)^19^; Thomas et al (GSE282122)^29^; Oliver et al (https://www.gutcellatlas.org)^21^; MSCCR (Argmann et al)^20^; plus microarray datasets: Ahrens et al (GSE10191)^63^; Gyorffy et al (GSE4183)^64^; Carey et al (GSE9686)^65^; Wu et al (GSE6731)^66^; and Kugathasan et al (GSE10616).^67^

**Table 2:** Patient Cohort Summary

Sex	Age range at sampling, *y*	Disease type	Tissue	Sampling method	Maximum modified Naini Cortana score	Maximum modified Reily score
F (123) M (104)	F (19–76) M (18–75)	CD (227)	Colon (78), ileum (149)	Resection (31)	11 (colon) 13 (ileum)	NA
				Biopsy (193) NA (3)	7 (colon) 7 (ileum)	NA
F (55) M (66)	F (19–67) M (18–79)	UC (121)	Colon (96), ileum (25)	Resection (24)	NA	19 (colon) 8 (ileum)
				Biopsy (96) NA(1)	NA	11 (colon) 4 (ileum)
F (20) M (26)	F (19–78) M (20–81)	non-IBD (46)	Colon (22), ileum (24)	Resection (7)	NA	NA
				Biopsy (39)	NA	NA

NOTE. Data are presented as number unless otherwise indicated.

CD, Crohn’s disease; F, female; M, male; NA, not available; UC, ulcerative colitis.

### Mouse Models and Experimental Intestinal Inflammation

C57BL/6J mice were purchased from Charles River and Janvier Labs and housed under specific pathogen-free conditions at Universitätsklinikum Erlangen. Glia reporter mice were generated by crossing *Plp1*^*CreERT*^^68^ with *Rosa26*^*tdTomato*^ mice, which carry a loxP-flanked stop codon upstream of tdTomato in the Rosa26 locus. *Plp1*^*CreERT*^ mice were also crossed with *Casp8*^fl/fl^ mice^69^ to generate *Casp8*^iΔGlia^. Genotyping for CreERT, tdTomato, and *Casp8*^fl/fl^ was performed by polymerase chain reaction (PCR) on tail DNA; primer sequences are available upon request.

*CreERT* recombination was induced by oral administration of tamoxifen (75 mg/kg body weight; Merck KGaA), solubilized in ethanol and diluted in corn oil (Merck KGaA), given once (glia reporter mice) or for 3 consecutive days (*Casp8*^iΔGlia^). Experimental groups included littermate controls housed in the same cages. Our experimental colitis and ileitis model databank was generated, sequenced, and analyzed using standardized protocols reported recently.^30^

Wild-type C57BL/6J or glia reporter mice were injected intraperitoneally with 30 μg of purified no azide/low endotoxin (NA/LE) hamster anti-mouse CD3e antibody (BD Bioscience) or hamster IgG NA/LE Isotype control (BD Bioscience) in phosphate-buffered saline (PBS), with a total volume of 200 μL. The course of inflammation was monitored by weight, clinical symptoms, and behavioral assessment, and sufficient enrichment and nesting material with ad libitum access to food and water was provided. Mice were sacrificed after 6 or 24 hours. Disease activity was scored for behavioral changes and clinical symptoms during the aCD3 model. A baseline was set right after the injection for the parameters weight, pilo erection, eyelid ptosis and facial swelling, and activity. Every 3, 6, and 24 hours, the mice were checked and given a score from 0 to 3 for the respective category. A total score for each mouse is reflected by the average of the score value of all categories. The percent weight loss was calculated for a score reflecting 0 to <5% weight loss as score 0, 5% to <10% weight loss as score 1, 10% to <20% weight loss as score 2, and ≥20% weight loss as score 3. Pilo erection, eyelid ptosis and facial swelling, and activity were score in increments of 0 to 3. The sample sizes were decided based on previous reports.^70^

For the motility assay, the mice were injected with 30 μg of purified NA/LE hamster anti-mouse CD3ε antibody (BD Bioscience) or hamster IgG NA/LE Iso control (BD Biosciences) in PBS, total volume 200 μL, intraperitoneally. After 2 hours, the food was removed from the cage, and the mice continued to be monitored. At the 5-hour mark, the mice received an oral gavage with 10,000 kDa fluorescein isothiocyanate–dextran (Merck KGaA) dissolved in 200 μL Lipofundin MCT20% (B. Braun Melsungen AG). After 6 hours, the mice were sacrificed via cervical dislocation, and the digestive tract from stomach to anus was removed. After short storage in ice-cold PBS, the intestine was laid out on a black background and imaged with the Amersham ImageQuant 800 detector. The intensity of the fluorescein isothiocyanate was measured and quantified with ImageJ and an in-house python script.

The generation, sample size estimation, cage groups, and analysis for the following models has been described in detail previously^32^: *Casp8*^ΔIEC^ ileitis and colitis, *Tnf*^ΔARE^ ileitis, acute and chronic dextran sodium sulfate–induced colitis, oxazolone-induced colitis, acute and chronic trinitrobenzene sulfonic acid–induced colitis, T-cell transfer colitis, *E vermiformis* infection, *Helicobacter hepaticus* infection, and *Citrobacter rodentium* infection.

Experimental protocols were approved by the Institutional Animal Care and Use Committee of the Regierung von Unterfranken and the Landesamt für Gesundheit und Soziales – Berlin, and in accordance with the UK Scientific Procedures Act of 1986. Generation and analysis of bulk transcriptomic data from the mouse models has described by us recently.^32^ The study reference numbers are as follows: 55.2.2-2532-2-1137, -1139, -1178, -1495, -1789; 55.2-2532-2-595, 55.2.2532–2-607, -712; G 0291/18; 54.2532.1.-15/12

### Ex Vivo “Plexanoid” Culture

Muscularis externa isolation and culture of all mature cells of the myenteric plexus (plexanoids) was adapted from established protocols.^54^^,^^71^^,^^72^ Littermates from C57BL/6J, glia reporter, *Casp8*^fl/fl^, or *Casp8*^iΔGlia^ mice were euthanized; the small intestine was extracted, flushed with ice-cold Hank’s Balanced Salt Solution (HBSS; no Ca^2+^/Mg^2+^; Gibco), and threaded onto sterile knitting needles (3–5 mm). Mesenteric fat and vasculature were removed, and the longitudinal muscle was incised and peeled using cotton swabs (Cobas Uni Swabs, Roche). Muscularis was cut (5-mm pieces), washed in ice-cold HBSS, and digested with collagenase type 4 (Worthington) in HBSS + 10% fetal calf serum (FCS) for 1 hour at 37 °C. Single cells were isolated with TrypLE (Gibco), triturated, and plated on Matrigel-coated plates. Culture was maintained in Neurobasal medium with 1% FCS, 1× B27, 1× glutamax, and penicillin/streptomycin. After 1 week, plexanoids were passaged 1:2 using TrypLE and replated on fresh Matrigel for further experiments.

### Stimulation of *Casp8*^iΔGlia^ Plexanoids

As described before, plexanoids of the *Casp8*^iΔGlia^ littermates were isolated and expanded, then replated on Matrigel (Corning) coated (diluted 1:30 in sterile PBS) 48-well cell culture plates. After 4 days of further proliferation in this setup, the medium was changed to neurobasal medium (Gibco) supplemented with Glutamax (Gibco) and penicillin/streptomycin (Merck KGaA) including a vehicle CTRL vs 50 ng/mL recombinant mouse TNF. For live cell imaging, 5 μg/mL of propidium iodide was added to the medium, and the vessels were monitored with the Incucyte live cell imager (Sartorius GmbH) for 72 hours.

### Cytokine Multiplex

The BioLegend LEGENDplex MU Th Cytokine Panel (12-plex) was used to reveal tissue cytokine levels. The tissue was homogenized, and the bead assay was performed as per manufacturer’s recommendations and instruction. In short, the samples were incubated with the capture beads in a V-bottom plate. After washing steps, the detection antibodies were added, and in a later step, conjugated to the fluorophore. After the final washing steps, the beads were analyzed in a BD Accuri C5 flow cytometer. The files were then uploaded to the BioLegend online tool and analyzed.

### Sorting, Culturing, and Stimulation of Enteric Glial Cells

Muscularis externa was isolated as described previously from glia reporter mice. Initially, cells were cultured as plexanoids to expand tdTomato^+^ glia. tdTomato expression was induced by adding 500 nM (Z)-4-hydroxytamoxifen (Merck KGaA) to the culture medium for 3 days. Single cells were obtained using TrypLE (Gibco) and resuspended in FACS buffer (PBS with 1% FCS and 2 mM EDTA). Sorting of tdTomato^+^ EGCs was performed at the FACS Core Unit, Friedrich-Alexander-Universität Erlangen-Nürnberg (FAU). Sorted cells were plated on Matrigel (1:30 in PBS; Corning) and grown in neurobasal medium (Gibco) with 1% FCS, 1× B27, 1× glutamax, penicillin/streptomycin (Merck KGaA), 20 ng/mL glial cell line–derived neurotrophic factor, and 20 ng/mL fibroblast growth factor–basic (Peprotech/ThermoFisher). For cytokine stimulation, cells were maintained in neurobasal medium supplemented with glutamax, penicillin/streptomycin, 20 ng/mL glial cell line–derived neurotrophic factor, and 20 ng/mL fibroblast growth factor–basic, with cytokines at 50 ng/mL (Table 3). Details on staining and antibody information is provided in Table 4.

**Table 3:** Cytokine and Other Stimulant Concentrations for EGC Cell Culture

Cytokine	Concentration	Catalogue #	Company
Ms TNF recombinant	50 ng/mL	12343016	ImmunoTools GmbH
Ms IFN-γ recombinant	50 ng/mL	575306	BioLegend, Inc.
Ms IL1β recombinant	50 ng/mL	12340013	ImmunoTools GmbH
Ms IL17A recombinant	50 ng/mL	12340174	ImmunoTools GmbH
Smac mimetic LCL161	5 μM	HY-15518	MCE MedChemExpress
ZVAD-FMK	20 μM	S7023	Selleckchem
Ms GDNF recombinant	20 ng/mL	450-44	PeproTech/ThermoFisher Scientific
Hu FGF-basic recombinant	20 ng/mL	100-18B	PeproTech/ThermoFisher Scientific

FGF, fibroblast growth factor; GDNF, glial cell line–derived neurotrophic factor; Hu, human; EGC, enteric glial cell; IFN-γ, interferon-γ; IL1β, interleukin1β; IL17A, interleukin17A; Ms, mouse; TNF, tumor necrosis factor; ZVAD-FMK, carbobenzoxy-valyl-alanyl-aspartyl-[O-methyl]-fluoromethylketone.

**Table 4:** Antibody Usage and Source Information

Antibody	Type	Dilution	Species	Code	Company
Primary antibody					
Anti-S100B	Unconjugated	1:2	Rabbit	GA504	Agilent Dako
Anti-TUBB3	Biotinylated	1:100	Mouse	801212	BioLegend, Inc.
Anti-GFAP	Alexa Flour 488	1:400	Mouse	644704	BioLegend, Inc.
Anti-CD4	Unconjugated	1:100	Rat	BD (553043)	BD Biosciences
Anti-pMLKL (S345)	Unconjugated	1:400	Rabbit	37333S	Cell Signaling Technologies
Secondary antibody					
Anti-rabbit	Biotinylated	1:500	Goat	111-065-144	Jackson
Anti-rat	Biotinylated	1:500	Goat	554014	BD Biosciences
Anti-rabbit	Alexa Flour 647	1:400	Donkey	406414	BioLegend, Inc.
Anti-rat	Dylight 550	1:400	Donkey	SA5-10027	ThermoFisher Scientific GmbH
FACS antibodies					
Anti-GFAP	Brilliant Violet 421	1:200	Mouse	644710	BioLegend, Inc.
MHC II I-A/E-A	FITC & APC-Cy7	1:2000	Rat	107606 & 107628	BioLegend, Inc.
Western blot antibodies					
PLP1	Unconjugated	1:500	Mouse	MCA839G	BIO-RAD
ACTB	HRP conjugated	1:2500	Mouse	ab49900	Abcam

FACS, fluorescence-activated cell sorting; FITC, fluorescein isothiocyanate; GFAP, glial fibrillary acidic protein; HRP, horseradish peroxidase; MLKL, mixed lineage kinase domain-like pseudokinase; PLP1, proteolipid protein 1; S100B, S100 calcium-binding protein B; TUBB3, tubulin, beta 3 class III.

For the induction of cell death, sorted EGCs were stimulated for 24 hours in reduced medium with IFN-γ (BioLegend, Inc), TNF (ImmunoTools GmbH), Z-VAD-FMK (Selleckchem), and combinations of these drugs (Table 3). For the live-cell imaging, CytoxGreen (Sartorius GmbH) was added according to the manufacturer’s recommendation. At the end of the experiment, the cells were used for immunostaining using anti-mouse phosphorylated Ser345 MLKL. For the immunostaining of the stimulated glia, the medium was exchanged to 4% paraformaldehyde (PFA) and incubated for 10 minutes at room temperature (RT). After washing twice with PBS, the cells were permeabilized with 1% TritonX (Merck KGaA) in PBS for 10 minutes at RT. Subsequent blocking was performed for 30 minutes at RT in PBS containing 1% bovine serum albumin (BSA; Carl Roth GmbH + Co. KG), 10% FCS (Merck KGaA) and 0.025% TritonX (Merck KGaA). The same buffer was used for the primary antibody (overnight at 4 °C) and secondary antibody (2 hours at RT) incubation with 2 PBS wash cycles after each incubation. Finally, Hoechst was added for nucleus counter staining for 10 minutes at RT. The images were acquired with a Leica SP5 confocal microscope.

### Flow Cytometry Analysis of Enteric Glia Population–Dependent Cell Death

In case of in vivo cell death FACS analysis, muscularis externa was stripped from intestines of tdTomato positive mice after 6 hours of aCD3 or Iso antibody injection as described before. The muscularis externa was then cut into small pieces and digested in Dulbecco's Modified Eagle Medium/F12 (Gibco) with Liberase TH (Roche), Dispase (Merck KGaA) and DNase (Merck KGaA) for 30 minutes at 37 °C. After following trituration in Dulbecco's Modified Eagle Medium/F12 (Gibco) with 2.5% BSA (Carl Roth GmbH + Co. KG) and 5 mM EDTA (Gibco), the cells were resuspended in FACS buffer (PBS with 1% FCS, 2mM EDTA) and filtered through 70- and 30-μm cell filters (Corning).

Subsequently, the respective cell solution was centrifuged at 400 rcf for 7 minutes at 4 °C. The supernatant was discarded, and the cell pellet was resuspended in FACS buffer. The different conditions were then stained with a fixable Live/Dead stain (ThermoFisher Scientific GmbH) and MHC-II antibody (BioLegend, Inc) for 45 minutes at 4 °C. After washing, cells were fixed in 4% PFA for 15 minutes at RT, washed again in FACS buffer, then stained for GFAP (BioLegend, Inc.). Flow cytometry was performed with the BD Fortessa system, and the data were analyzed in FlowJo v10.10.

### Hematoxylin and Eosin–Stained Paraffin Sections of Intestinal Tissue Were Used for Muscle Thickness Measurements

The muscle thickness was defined as the perpendicular distance from the bottom of the crypts to the outer gut wall and was measured using the NDP.view2 software. Areas containing lymphoid follicles and Payer’s patches or where the section ran longitudinal to the axis of the gut were excluded from the analysis. All clearly defined crypts were chosen, and the final quantification is based on average distances from 1 section with an average of ∼40 crypt–muscle measurements per mouse. Statistics are drawn on biological replicates, not on number of crypts.

### Immunohistochemistry and Immunofluorescence Staining

For the hematoxylin and eosin (H&E) staining, we prepared sections of 5- to 10-μm from tissues fixed in formalin and embedded in paraffin, which were then subjected to standard H&E staining protocol. H&E images were obtained using a NanoZoomer 2.0 (Hamamatsu).

The H&E scoring for bleeding is defined by the amount of red blood cells clearly outside of blood vessels and in the lumen of the intestine. The H&E sections were taken from the same duodenal region for each animal, and the Iso control animals used for baseline comparison. The scoring of Casp8flox animal and Casp8iΔGlia treated with anti-CD3 was performed blind with anonymized filenames.

The immunostainings were performed on paraffin sections of the tissues, which were cut, deparaffinized, hydrated, and treated with a Tris-EDTA-based antigen retrieval solution. To prevent nonspecific binding, a commercial blocking reagent (Immunoblock 1X, Carl Roth GmbH + Co KG) was utilized. The tissue sections were then incubated overnight at 4 °C in the dark with the primary antibody for each immunostaining (Table 4). We washed the samples in tris-buffered saline, and then we incubated them with either the secondary antibody or streptavidin conjugates (DyLight, ThermoFisher Scientific GmbH) for 1 hour at 4 °C. Samples then were counterstained with Hoechst 33,342 (ThermoFisher Scientific GmbH) for the cellular nuclei and cover-slipped in fluorescence mounting medium (Agilent Dako). A Leica TCS SP5 confocal microscope (Leica Microsystems) was employed for the acquisition of the immunofluorescence images using the necessary settings.

### Whole Mount Immunostaining

Whole mount staining was performed as previously described, with some modifications. Intestinal tissue was obtained from duodenum and jejunum in approximately 1-cm length, opened longitudinally, and placed between 2 microscopy slides. These slides were submerged in 4% ice-cold PFA and carefully agitated for no longer than 2 hours. After washing the tissue twice with PBS, 0.025% Triton X (Merck KGaA) and 0.02% Tween20 (Merck KGaA) in PBS was used to permeabilize the tissue. After blocking unspecific binding side with Roti Histoblock (Carl Roth GmbH + Co. KG) in 0.025% Triton X (Merck KGaA) and 0.02% Tween20 (Merck KGaA) in PBS, primary antibodies were incubated for 7 days at 4 °C in a blocking solution with Roti Histoblock (Carl Roth GmbH + Co. KG), 0.0125% Triton X (Merck KGaA), 0.01% Tween20 (Merck KGaA), and 0.01% sodium azide (Carl Roth GmbH + Co. KG) in PBS. After subsequent washing steps with PBS, secondary antibodies were incubated in blocking solution for 4 days. After final washing steps in PBS, tissue was stored in PBS containing 0.02% sodium azide (Carl Roth GmbH + Co. KG). For imaging, the tissue was placed between 2 coverslips with mounting medium. Imaging was performed on a Leica confocal microscope SP5.

### RNA Isolation, Reverse Transcription, and Quantitative Polymerase Chain Reaction

RNA was obtained from homogenized tissue or FACS-sorted EGCs with the column-based RNeasy kit (Macherey-Nagel GmbH). After quality control via Nanodrop, 200 ng of the RNA was processed into first-strand complementary DNA using the reverse transcriptase kit (Jena Bioscience GmbH) according to manufactures instructions. For quantitative PCR, 2 μL of complementary DNA was used in a 20 μL SYBR Green based PCR reactions in 96-well format, reaction performed in a BioRad PCR CFX Connect OM.

### Bulk Messenger RNA Sequencing

RNA was obtained as described before. RNA quality control was performed with Nanodrop analysis and Agilents TapeStation automated electrophoresis. Between 100 to 200 μg of total RNA samples were shipped to and analyzed by Novogene GmbH, Munich, Germany, including a secondary quality control and library preparation. Sequencing results were analyzed with an in-house pipeline described earlier.^30^

### Western Blotting

Protein samples were prepared from snap-frozen tissue. The samples were put in ice-cold T-Per lysis buffer (Merck KGaA) with protease and phosphatase inhibitors (Roche, 1 tablet per 10 mL) and 1% phenylmethylsulfonyl fluoride (Roche). With a steel bead added to the tissue, the sample was homogenized in a high-speed shaker (Retsch GmbH) at 25 Hz for 2 minutes. Further lysis was performed on ice for 20 minutes and subsequent centrifugation for 20 minutes at 4 °C at 14,000 rpm. Protein concentrations from the supernatants were determined via standard Bradford method and equal amounts of protein per sample were mixed with 4× loading buffer containing 40 mM 1,4-dithiothreit (Carl Roth GmbH + Co. KG). The samples were boiled at 95 °C for 5 minutes and loaded on a precast sodium dodecyl sulfate-polyacrylamide gel electrophoresis gels (BIO-RAD).

Gels were run in BioRad electrophoresis chambers at 200V for approximately 45 minutes. The gel was transferred on polyvinylidene fluoride membranes with the BioRad Turboblotter and membranes blocked with 5% milk powder (Merck KGaA) for 1 hour at RT. Subsequently the membranes were probed for the target proteins with primary antibodies (Table 4) diluted in 5% BSA (Carl Roth GmbH + Co. KG) in TBST overnight at 4 °C. Secondary antibodies coupled with horseradish peroxidase are added after washing and incubated for 2 hours at RT. Signal detection with Western Lightning Plus ECL (Revvity, Inc) was done with the Amersham ImageQuant 800 detector. Total change of proteins was normalized to β-actin. Membranes that were probed for more than one protein were stripped-off the bound antibodies with a gentle stripping buffer consisting of 1 g/L sodium dodecyl sulfate (Carl Roth GmbH + Co. KG), 15 g/L glycine (Carl Roth GmbH + Co. KG), 10 mL of Tween 20 (Merck KGaA), and brought to a pH of 2.2 with 32% HCl (Carl Roth GmbH + Co. KG). The membranes were shaken in the gentle stripping buffer twice for 10 minutes at RT, washed with PBS and TBST for a total of 4 times for 5 minutes, 2 times, respectively.

### Combined Cell Death Signature Gene List

To track the changes in cell death signature genes, we used a combined approach tracking human and mouse genes belonging to the Kyoto Encyclopedia of Genes and Genomes pathways for both apoptosis and necroptosis. Several of these genes share regulatory behavior and tend to be coregulated with each other. Therefore, we generated the combined cell death signature list from the pathway gene lists shown in Table 1 below. The average expression of these genes was tracked on the respective human and mouse snRNA-seq and scRNA-seq datasets.

### Plot Generation Software

Data was analyzed in commercial software GraphPad Prism program (Version 10) or in-house python (Version 3.11.0) and R scripts (Version 4.4.0). For heatmaps, the ComplexHeatmap (Version 2.20.0) package was used, Ridge plots were generated using the ggridges package (0.5.6). Other plots in R were generated using the ggplot2 package (3.5.1). Matplotlib (3.9.0) and Seaborn (0.13.2) were used for the figure plots generated using python (3.11.0). Other packages used for data analysis and visualization are listed in Table 5 below.

**Table 5:** Software Packages and Versions Used in Data Analysis and Visualization

Package name	Version
R version 4.3.3	
ggplotify	0.1.2
progeny	1.24.0
ggfortify	0.4.16
RColorBrewer	1.1-3
ComplexHeatmap	2.18.0
ggrepel	0.9.5
scales	1.3.0
openxlsx	4.2.5.2
tidyr	1.3.0
ggplot2	3.4.4
cowplot	1.1.2
DESeq2	1.42.0
extrafont	0.19
fmsb	0.7.6
dplyr	1.1.4
tidyverse	2.0.0
ggridges	0.5.5
decoupleR	2.8.0
gridExtra	2.3
circlize	0.4.16
Python 3.12.3	
numpy	1.26.2
pandas	2.0.3
scanpy	1.10.4
anndata	0.11.1
matplotlib	3.8.2
scipy	1.11.4
seaborn	0.13.2
scipy	1.11.4
statsmodels	0.14.4
igraph	0.11.8

### Single-Nucleus RNA Sequencing of Formalin-Fixed Paraffin-Embedded Tissue Samples

Human formalin-fixed, paraffin-embedded full-thickness intestinal wall sections from 2 CD, 4 UC, and 5 non-IBD CTRLs were processed using the 10x Genomics Single Cell Gene Expression Flex Kit (‘CG000632_RevC’). Briefly, tissues were deparaffinized, rehydrated, and dissociated in ice-cold PBS with enzyme mix using a pellet pestle. After trituration and filtration (30 μm), single nuclei were collected and counted. After quality control of the nuclei, probe hybridization was performed in bulk, followed by gel emulsion microbead encapsulation using 10x Genomics droplet-based cassettes. Nuclei were packed into gel emulsion microbeads with the 10x Genomics droplet-based single cell cassette. Next, probes were ligated to nuclei and barcoded to enable sn identification. Library prep followed the ‘CG000527_Rev_E’ (multiplex) or ‘CG000691_Rev_A’ (singleplex) guides, targeting ∼10,000 nuclei/sample. Sequencing was done at the MDC-BIMSB Genomics Core (Illumina NovaSeq X), targeting 20,000 read pairs per nucleus.

Libraries were demultiplexed with bcl2fastq (Illumina), and FASTQs processed with Cell Ranger (v7.1.0, 10x Genomics; GRCh38-2020-A reference). Data analysis was performed in R (v4.3.3) using Seurat (v5.0.1). Ambient RNA was removed (SoupX v1.6.2), and nuclei filtered (>400 genes, >450 unique molecular identifiers, <15% mitochondrial, <5% hemoglobin reads). Doublets were excluded (DoubletFinder v2.0.4), and merged data further filtered (>4000 genes, >10,000 unique molecular identifiers). Standard Seurat workflows (log normalization, scaling, Principal Component Analysis, Uniform Manifold Approximation and Projection using 35 PCs) were applied. Harmony (v1.2.0) corrected for batch effects; followed by SNN-graph–based clustering (resolution = 0.25). Initial annotation relied on canonical markers (epithelial: EPCAM, KRT8; stromal: CDH5, COL1A1/2; immune: PTPRC, CD3E, CD79A). Subclustering refined annotations within each compartment. For this study, EGCs were identified using canonical glial markers (SOX10, PLP1) and extracted for dedicated preprocessing and analysis. Details of software packages used are provided in Table 5.

### Statistics

Bulk RNA-seq data was presented as gene-wise scaled counts and nonparametric statistical tests (Mann–Whitney tests). In cases where log normalized fold changes were derived against respective control groups using DESeq2, the Wald statistic associated with DESeq2 was used. For comparing groups from the analysis of sc and sn data, Benjamini–Hochberg false discovery rate corrected *P* values derived from the 2-tailed Mann–Whitney *U* test vs respective controls are shown. Normal distributions were not assumed in mouse experiments. For column-wise comparisons of over 2 groups, 1-way analysis of variance followed by Kruskal–Wallis post-hoc test was used and for grouped data analysis was performed using 2-way analysis of variance followed by Tuckey’s post-hoc test. Alternative post-tests applied for time course data have been indicated directly below the corresponding figures. The respective *P* threshold of .05 was used throughout to call significance.

## Supplementary Material

Supplementary Material

Extended PDF
