# Supplementary material for: Coordinated Interferon-γ/Tumor Necrosis Factor Axis Drives Selective Loss of Activated Enteric Glia in Inflammatory Bowel Diseases
Source: Cell Mol Gastroenterol Hepatol. 2026 Jun 12;20(10):101827. doi: 10.1016/j.jcmgh.2026.101827 (PMC13452377; doi:10.1016/j.jcmgh.2026.101827)

Full-size Western Blots. Related to supplemental Fig. 3F

Lanes 7 to 12 are not related with this article

PLP1: MW 20-30 kDa, the lower band is an alternative splice form of PLP1 known as DM20.

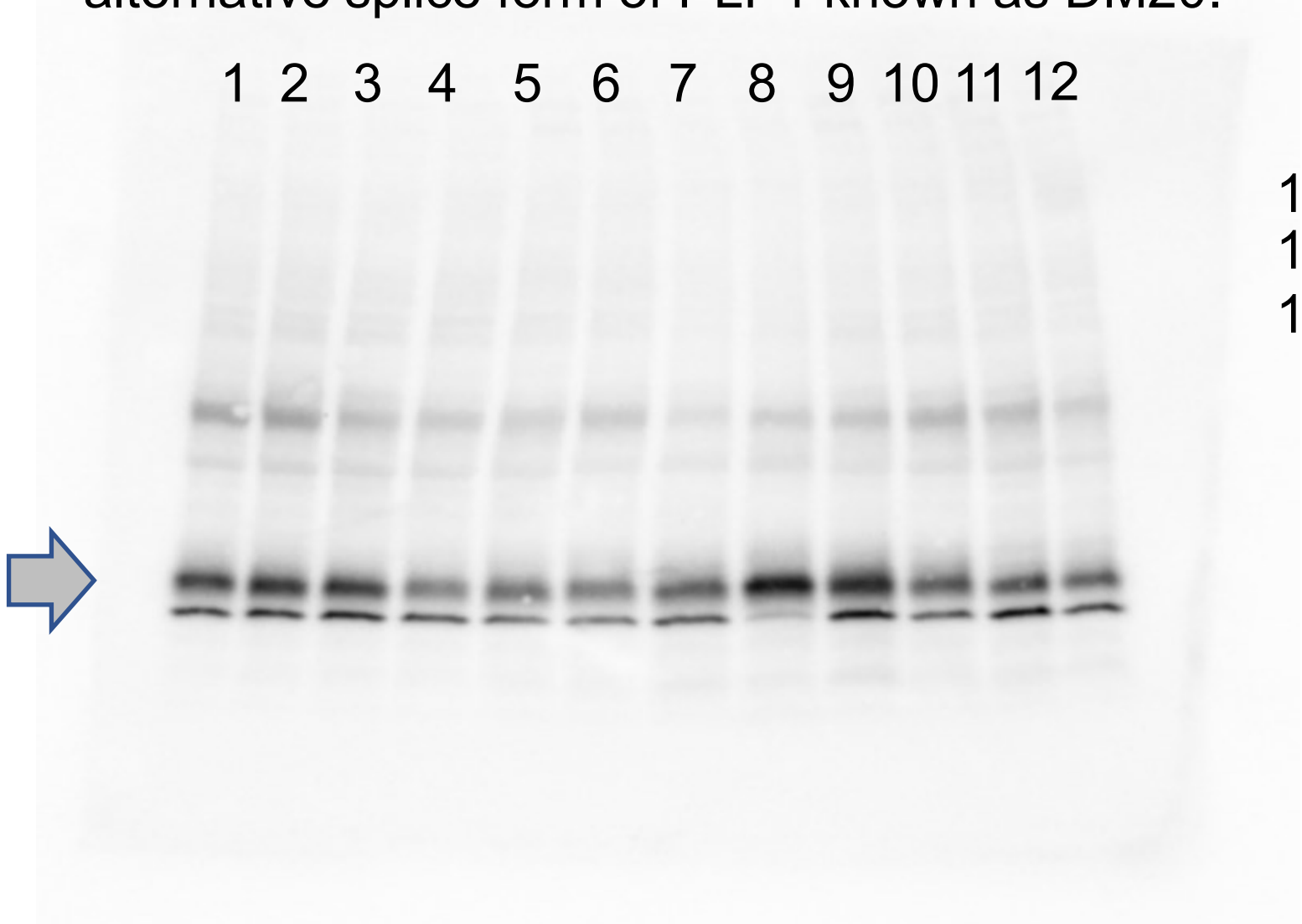

Oversaturated overlays with the protein ladder. Numbers to the left indicate MW of the ladder

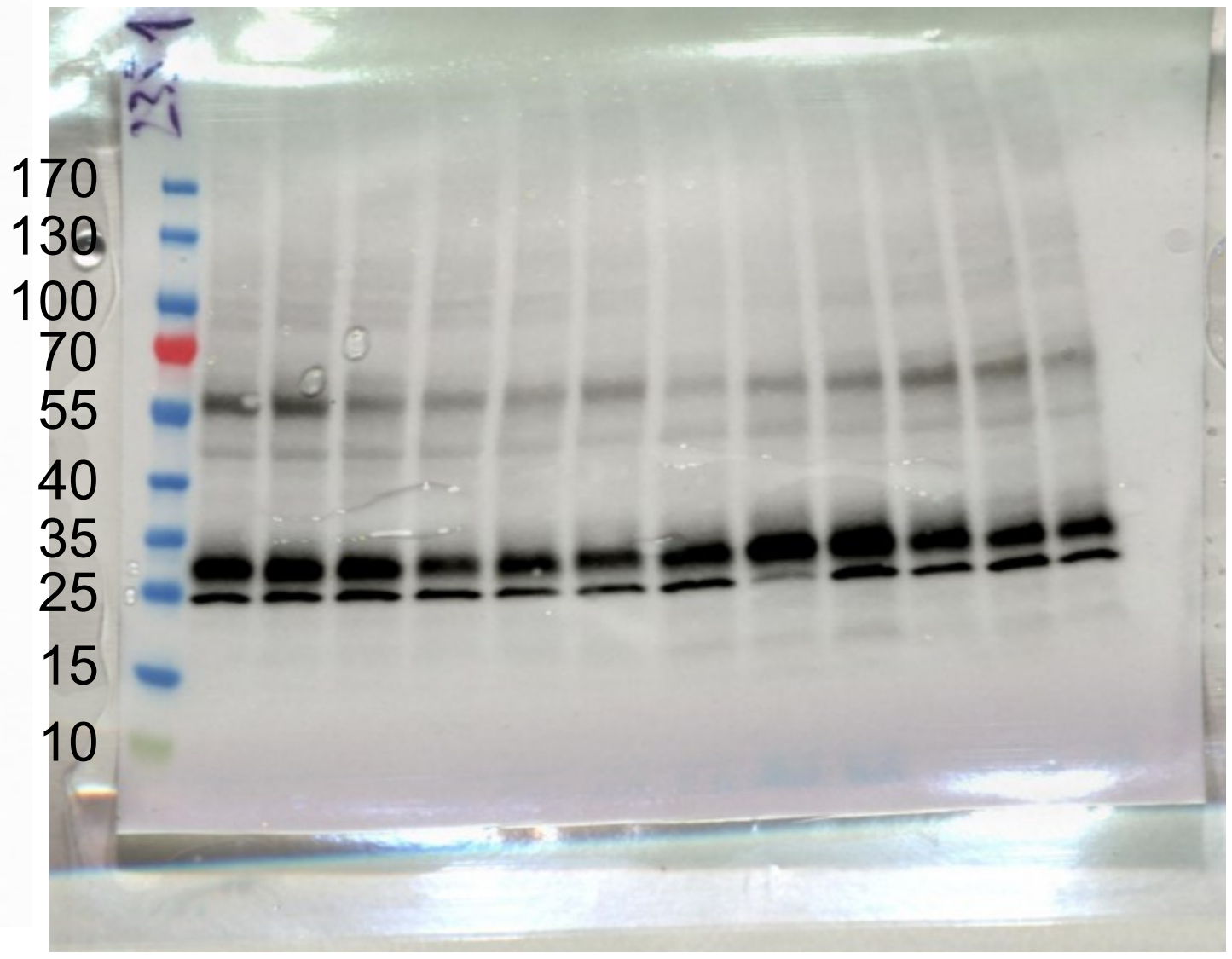

β-actin: MW 45 kDa

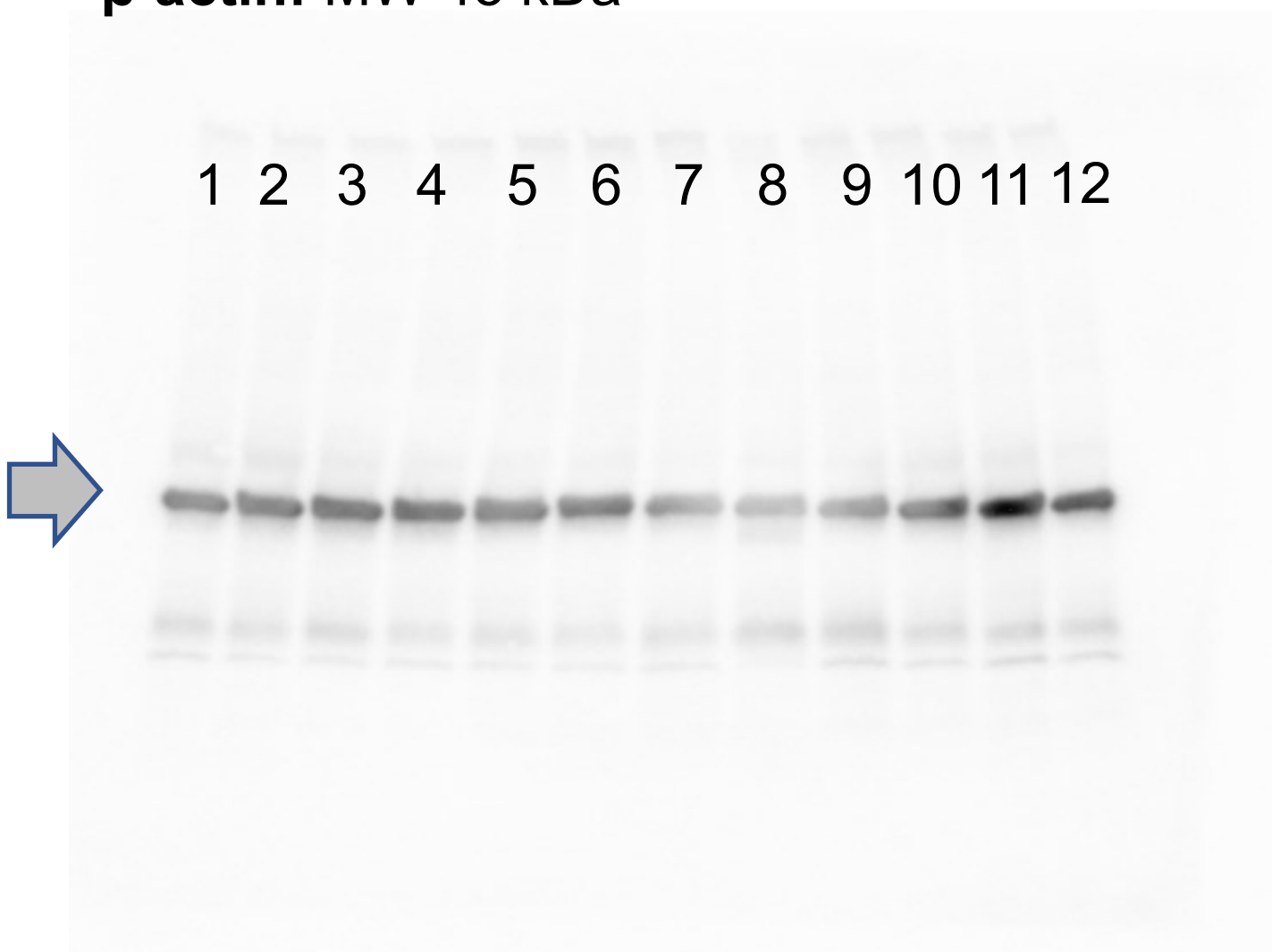

Oversaturated overlays with the protein ladder

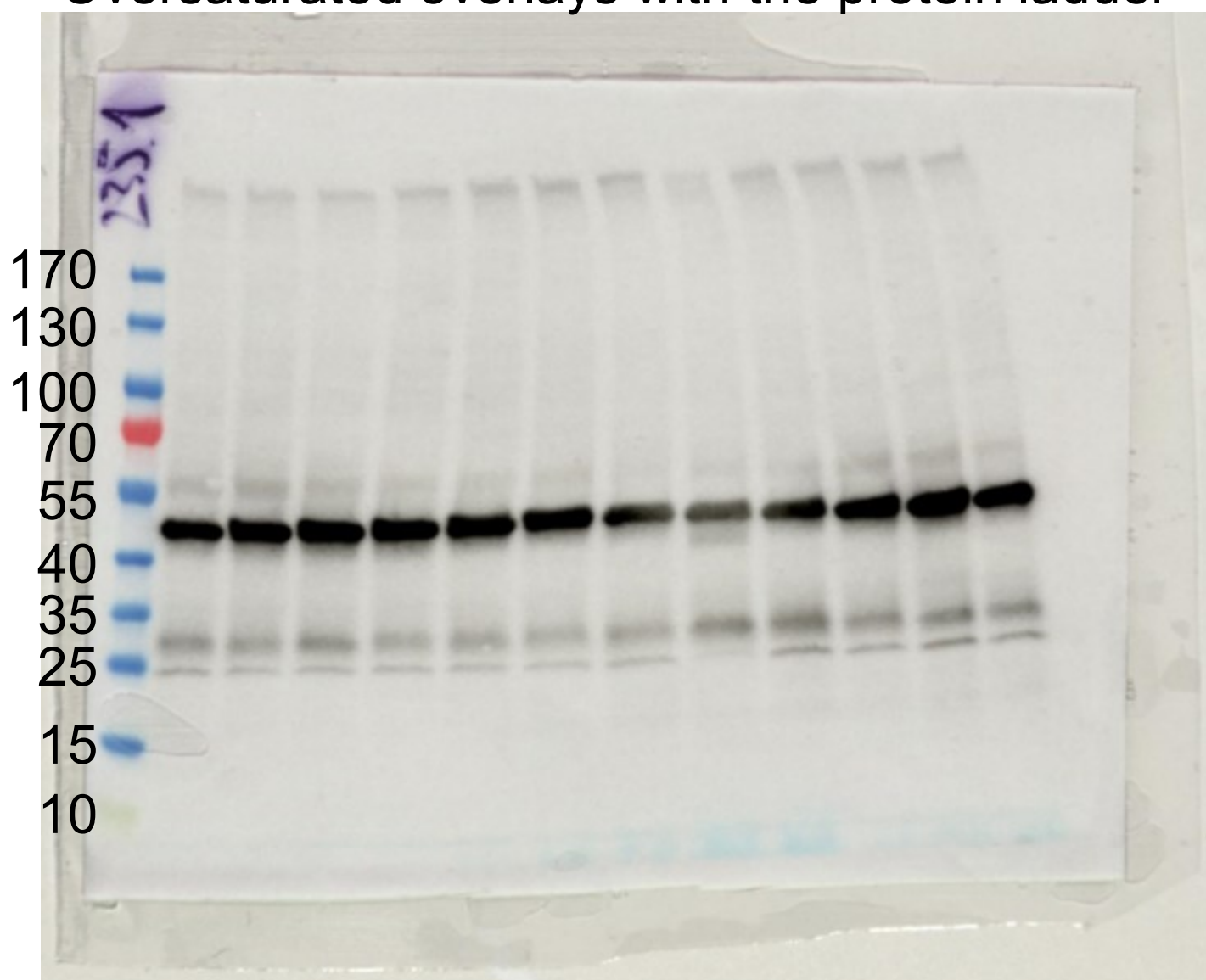

Supplement: Supplementary file 2 [file mmc2.pdf]
